# Towards net zero land biotechnology: an assessment of biogenic feedstock potential for selected bioprocesses in Germany

**DOI:** 10.1186/s13068-025-02673-y

**Published:** 2025-07-09

**Authors:** Adrian Tüllinghoff, Heike Sträuber, Flávio Cesár Freire Baleeiro, Andreas Aurich, Micjel Chávez Morejón, Kathleen Meisel, Karl-Friedrich Cyffka, Falk Harnisch, Katja Bühler, Daniela Thrän

**Affiliations:** 1 Department of Microbial Biotechnology, Helmholtz-Center for Environmental Research-UFZ, Permoserstr. 15, 04318 Leipzig, Germany; 2 Department of Systemic Environmental Biotechnology, Helmholtz-Center for Environmental Research-UFZ, Leipzig, Germany; 3 DBFZ-Deutsches Biomasseforschungszentrum gGmbH, Leipzig, Germany; 4 Department of Bioenergy, Helmholtz-Center for Environmental Research-UFZ, Leipzig, Germany

**Keywords:** Biogenic residues, Circular bioeconomy, Biotechnology, Residue-based production, Land use

## Abstract

To stay within the planetary boundaries circularizing economy by utilizing residues is key. Bioprocesses can use abundant, but complex biogenic residues, giving access to various value-added products. To advance circularization, the feasibility of exploiting diverse biogenic residues as feedstocks for different, yet specific, bioprocesses needs to be assessed. Exemplifying the national level in Germany, we categorized biogenic residues compiled in the DE Biomass Monitor regarding their composition and feedstock potential in a resource matrix, detailing their constituents and the quality of available data. Three biotechnological processes, making use of lignin, non-fibrous carbohydrates, and oil, respectively, served as model processes to assess the biogenic production potential. By developing material flows based on state-of-the-art conversion routes, we found that residue-based production via all three example processes could meet national demands of specific polymer bricks, medium chain carboxylates, and platform chemicals, respectively, when mobilizing only 20–30% of possible raw materials. The accruing side streams underline the importance of cluster approaches early in bioprocess development. Specific challenges for fully exploiting the potential of biogenic residues were identified, including legal and acceptance issues, the need for considered biomass decomposition in interweaved production lines, and residue availability and management. This study provides an example-based framework for integrating biogenic residues with biotechnological production, using the resource matrix and an initial material-to-product estimation to advance a circular bioeconomy.

**Supplementary Information:**

The online version contains supplementary material available at 10.1186/s13068-025-02673-y.

## Highlights

- Comprehensive compilation of 35 biogenic residues in a resource matrix
- Identifying suitable residues and material flows for three exemplary bioprocesses
- Estimating production potential and land use savings for residue-based production

**Supplementary Information:**

The online version contains supplementary material available at 10.1186/s13068-025-02673-y.

## Introduction

On a global level, mankind today consumes the 1.7-fold of the resource amount that sustainably can be restored [1]. To stay within our planetary boundaries, circularity quotes have to be dramatically increased: in Germany, the circular material use (CMU) rate was only 12.2% in 2019, which is below the EU average (12.4%) and lacks far behind countries, such as France (20.1%), Belgium (24.8%), and the Netherlands (28.5%) [2]. In particular, the CMU of biomass ranges below national average with 7.6% in 2019 and did not show any sign of consistent increase within the last decade [3]. Yet, nature-based resources are especially crucial for achieving a truly circular bioeconomy. In this envisioned scenario, where competition for arable land will be fierce, net zero land solutions are of crucial interest.

In this context, utilizing biogenic residues (s. Glossary in the SI), including biogenic by-products and wastes [4], as feedstock for biotechnological production is an ideal approach that does not compromise food and feed security. Biotechnological production processes are ideal for exploiting biogenic residues, as compared to petrochemistry, they are generally perceived as more sustainable, as they are assumed to be more resource efficient and energy saving [5–7]. Due to the typically mild reaction conditions in terms of temperature, pressure, and use of (mostly) water-based solvents, biotechnological processes meet 9 out of 12 principles of green chemistry [8]. Despite promising attempts to use flexible feedstock in industrial biotechnology [9], the extent to which available residues can be utilized in newly developed bioprocesses remains unclear: while data on the amounts of accruing biogenic residues are accessible, detailed information on biochemical composition is not directly available.

In a recent endeavor, the biogenic residues accruing in Germany were investigated for the first time and published in the DE Biomass Monitor [10–13]. Here, 77 biogenic resources from five sectors, including agriculture, forestry, industry, and municipal waste, are compiled with their annual tonnages in an accessible format [12]. The database reveals the overall technical potential of biogenic residues of more than 100 Mio Mg (1 Mio Mg equals 10^6^ t) dry matter (DM) per year in Germany [10], which is estimated to be about a tenth of biogenic residues accruing in the European Union [14]. The technical potential is defined as the amount of biomass which is available using current technologies, taking into account spatial restrictions due to competition with other land uses, such as food, feed, and fiber production, as well as non-technical constraints [15]. The biogenic residues range from various types of straw and other agricultural wastes, such as manure, over forestry by-products, such as wood shavings and black liquor, to residues from various industries and municipal wastes, such as bio-waste from private households and wastewater. Thus, they differ largely in terms of physico–chemical properties, biochemical composition, quality, and temporal and spatial availability. To mobilize this enormous technical potential, the prospective resources have to be screened for their feasibility to serve as feedstock for biotechnological production.

Biomass is composed mainly of cellulose, hemicellulose, lignin, non-fibrous carbohydrates (NFC), proteins, fats and oils, and inorganic compounds, often referred to as ash [16, 17]. The shares of these fractions even within the same biogenic residue vary considerably depending, e.g., on its source, storage, and treatment. While there are studies on the biochemical composition of resources from a certain type or sector, such as agriculture and forestry [18], examples of a likewise assessment crossing the sectors are rare and typically only consider few resources [19]. Some specific biomass fractions are important resources in established industry or agriculture, e.g., cellulose in pulp and paper production and protein-rich biomasses for animal feed [20, 21]. Furthermore, hemicellulose is easy to mobilize and can be converted to platform chemicals [22] or biofuels, such as ethanol [23, 24] by means of biotechnology. Here, recent advances enable flexibilization of feedstock beyond the conventionally used biomass fractions [9]. Typically, the different bioprocesses are based on one specific biomass fraction thwarting the product yield. Combining multiple process lines in a cluster approach will be key to utilize all biomass fractions [22], and thus use biogenic feedstock to its full potential.

In this study, we set out to answer the question to which extent available residues can be used as feedstock for bioprocesses and which product amounts could be achieved. To this end, we selected three model processes described in brief below converting biomass fractions that typically are not used for material production specifically being polymer bricks from lignin, carboxylic acids from non-fibrous carbohydrates (NFC) and tricarboxylic acid (TCA) cycle intermediates from waste cooking fat and oil (WFO). These model processes are presented according to their technology readiness level (TRL) from being feasible in lab scale (TRL 2/3) to first technology demonstrations (TRL 6).

In the first model process, polymer bricks can be produced from lignin. The lignin fraction is mobilized by a two-step process, combining electrochemical and microbial conversion [25]. Phenolic monomers from decomposed lignin macromolecules are electrochemically reduced to hydrogenated intermediates, such as cyclohexanol, which subsequently are converted by engineered *Pseudomonas taiwanensis* VLB120 to produce adipic acid [26]. As prime example adipic acid makes up approximately 50 mol% of Nylon-6,6, and has an annual production of 4.5 Mio Mg [27].

Furthermore, NFCs are a readily fermentable fraction of biomass that, along with portions of cellulose and hemicellulose, can be digested in biogas reactors to produce biomethane. By inhibiting methane formation during the anaerobic digestion of biomass, short- and medium-chain carboxylic acids and H_2_ are produced [28]. The conversion of the NFC fraction has particularly high selectivity to caproic (C6) and caprylic (C8) acid [29], which are relatively easy to extract from the fermentation broth and have uses in animal nutrition, fragrances, flavors, food ingredients, lubricants, and other markets [30]. C6 and C8 carboxylic acids are estimated to have a total market size of about 1.8 Mio Mg a^−1^ as of 2024 [31]. Capraferm^®^ (carboxylic acids from NFC fermentation) is one of the contender processes for the production of medium-chain carboxylic acids from carbohydrate-rich feedstock that is currently being scaled up in Germany [29, 30, 32]. The microbial communities used in this process are also able to convert syngas (i.e., H_2_, CO_2_, and CO) mixotrophically [33], which opens up the possibility of coupling carboxylic acid production with CO_2_ fixation.

In a third model process, tricarboxylic acid (TCA) cycle intermediates are produced from waste cooking fat and oil (WFO). The fat and oil fraction is a highly relevant feedstock for biodiesel production. In terms of material production, microbial conversion with the yeast *Yarrowia lipolytica* gives access to important platform chemicals related to intermediates of the TCA cycle, such as citric acid (CA) [34], which are of higher value than biodiesel. CA is an excellent example of a bio-based commodity with an annual production of 2.0 Mio Mg a^−1^ [35]. Many established applications of CA are known, ranging from its use as cleaner, decalcifier, acidulant and stabilizing agent, via use in personal care products, animal feed up to applications in metallurgy and plant protection [36, 37]. Meanwhile, raw glycerol, a by-product of biodiesel production and the backbone molecule of triglycerides, can be converted to α-ketoglutarate (KGA) by *Y. lipolytica* [38]. KGA has versatile applications, especially as a polymer building block (elastomers, bioactive N-heterocycles) and for crop protection [39].

To integrate biogenic residues as feedstocks for biotechnological production processes and assess their potential several prerequisites need to be met. While data on the biochemical composition of specific biogenic residues is available, a coherent, sector-crossing database reflecting the diversity of available resources is missing. To bridge this gap, we categorized the biogenic residues listed in the DE Biomass monitor in terms of their biochemical composition. Compiling data in a resource matrix allowed us to address the core question which residues are suited for which (type of) bioprocess. To answer the follow-up question, which process steps are required to mobilize the potential of a specific biogenic residues to its full extent, possible material flows were set up for the three model processes introduced above. These material flows served as a basis to illustrate achievable product amounts based on biogenic residues (Figure S1). Finally, general conclusions for an envisioned circular bioeconomy in a European framework are discussed.

## Materials and methods

To assess the potential of biogenic residues for their use as feedstock for biotechnological processes, the categorization of biomasses according to the DE Biomass Monitor was used (Section "Resource categorization").

The biochemical composition of a specific resource is of particular interest for evaluating its feedstock potential. In the first part, the biochemical composition of the most relevant resources was determined based on recent literature (Section "Biochemical composition of biogenic residues"). The data quality for each resource was assessed and described by a quality score (Section "Data quality level").

In the second part, a resource estimation was conducted. Based on the biochemical composition of the individual resources, the biogenic production potential of the three different model processes was calculated (Section "Data quality level").

### Resource categorization

For the cross-sector description of the raw material basis, the individual biomass types need to be unambiguously identifiable [12]. We, therefore, used the biomass residue categorization established by [11] that sorts a total of 77 biogenic residues based on their origin. Thus, the biogenic residues are categorized in the five sectors agriculture, forestry, industry, municipal waste and sewage sludge, as well as residues from other sectors. Following this categorization, the individual biogenic residues were described in terms of their biochemical composition (Section "Resource categorization") and the provided data quality (Section "Biochemical composition of biogenic residues").

### Biochemical composition of biogenic residues

For the mobilization of biogenic residues as feedstock, detailed and reliable data on the biochemical composition of each resource is essential. Respective information is not comprised in the DE Biomass Monitor. Thus, literature search was conducted to compile information on the biochemical composition, i.e., the fractions of cellulose, hemicellulose, lignin, NFC, protein, fats and oils, and ashes in each raw material. Detailed information about the literature can be found in the Supporting information right after the glossary (see also Table S1).

As an easily accessible criterion for the relevance of a resource, the technical potential was chosen. It is defined as the available amount of a resource under given technical restrictions, taking into account spatial and other non-technical constraints [15]. For the technical potential, the most recent data were extracted from the DE Biomass monitor for each resource [10]. Considering the overall amount of > 100 Mio Mg a^−1^ biogenic residues, all resources with a technical potential below 250,000 Mg per year were excluded. This reduced the portfolio of the screening from 77 to 35 residues, but provided detailed information on the biochemical composition of a total from 98% from literature with respect to technical potential listed. The obtained data were arranged in a resource matrix interlinking the biogenic residues with their biochemical composition. For each residue and each fraction, the minimum (MIN), average (MEAN), and maximum (MAX) values are listed.

Information on pollutants and inhibitors are considered to be of interest for the assessment of the usability of biogenic resources as feedstock. However, given the multitude of biotechnological methods and processes, general statements cannot be made and hence pollutants and inhibitors are not included in the resource matrix.

### Data quality level

Data on the biochemical composition are compiled from very heterogeneous sources. To give a general assessment, the data quality on biochemical composition of screened residues was evaluated according to [12] with some modifications. Five quality levels were established based on the criteria (i) number of related sources; (ii) quality of analysis and measuring scope; and (iii) recency. Each level was color-coded from green (1, good data quality) to pink (5, not reliable quality). Table 1 presents the specific requirements for each data quality level. Screened residues were given the best (= lowest) score, for which they meet all three criteria. For instance, a residue with more than 2 related sources from 2010, containing detailed information on analysis available including a series of measurements, would be scored with 3 (= sufficient), as the recency does not allow for a better data quality score. The number of related sources (> 2, > 1, or 1) and recency (< 5 years, < 10 years, < 20 years, or < 30 years) are directly quantifiable criteria. The quality of analysis incorporates both the accessibility of information about the used methodology and the scope of the measurements.

**Table 1:**
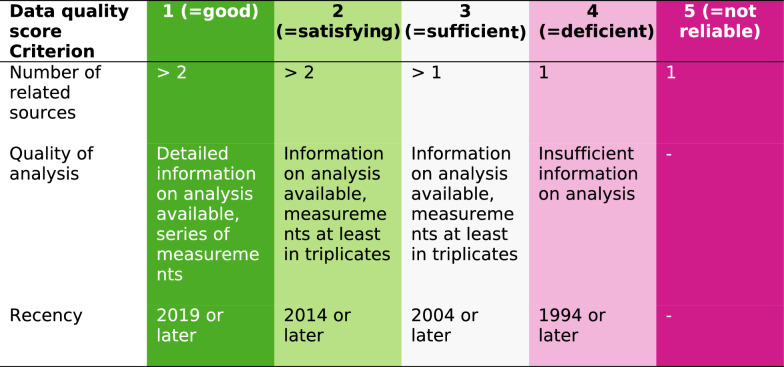
Five level system of data quality. Each requirement of the three criteria on the left has to be met to be evaluated with a certain data quality score

### Resource potential assessment for the model processes

For the three model processes, the biogenic production potential based on the resource matrix was estimated. Favorable raw materials for each process were selected based on a high share of the relevant fraction to be converted. For the production of polymer bricks, medium-chain carboxylates, and CA, a lignin content of ≥ 20%, an NFC content of ≥ 16%, and an oil/fat content of > 90% (all % DM), respectively, based on technical feasibility of the respective processes [40–42].

**Fig. 1:**
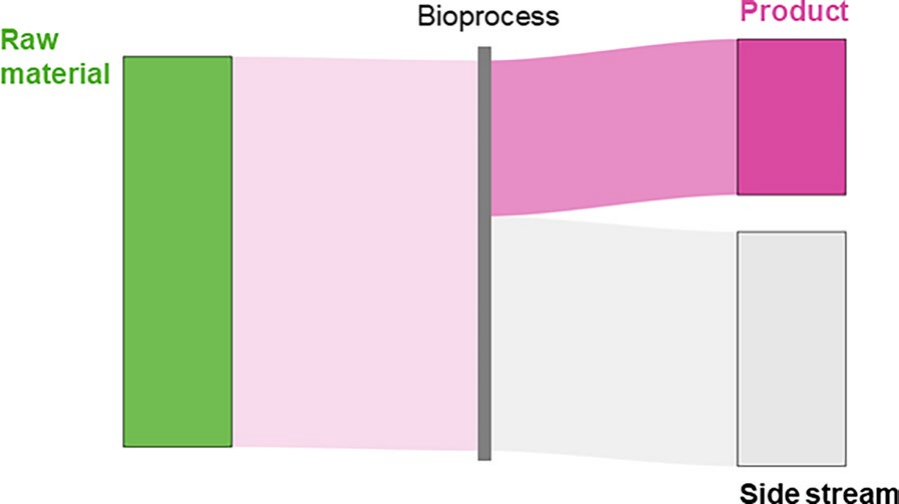
General material flow scheme. Raw material is converted in a bioprocess yielding product and side-stream. The bioprocess can include several steps

A model bioprocess was defined as the conversion of raw material into product and side stream (Fig. 1), typically consisting of individual process steps. Depending on the type of bioprocess, these steps include pre-treatment (e.g., biomass fractionation and depolymerization) and production (e.g., electrochemical and microbial conversion). The envisioned process flows of all model processes are illustrated in Figure S2. The side stream is the share of a (processed) raw material not converted into the product, but typically constitutes a valuable resource for other processes.

Conversion factors and yields for individual process steps are derived from technical and scientific literature on state-of-the-art methods (Table 2). They cover preparation of raw material, such as decomposition and depolymerization, as well as conversion factors of the respective bioprocesses. It has to be noted, that the considered studies are highly different in terms of TRL.

**Table 2:** Resource requirements and process parameters of the model processes

Model process	Process step	Input	Product	Conversion factor^a^	Refs.
Polymer bricks from lignin	Biomass decomposition	Lignin-rich residues	Lignin fraction	0.59	[22]
	Lignin depolymerization	Lignin fraction	Phenolic monomers	0.35 (0.50^c^)	[43, 44]
	Electrochemical conversion	Phenolic monomers	Cyclohexanol (incl. derivatives)	0.68 (0.85^c^)	[25]
	Microbial conversion	Cyclohexanol (incl. derivatives)	Adipic acid	0.61 (0.99^c^)	[25]
Carboxylic acids from NFC	Microbial conversion with Capraferm®	NFC-rich residues	C_6_/C_8_-carboxylic acids	0.093^b^	[32]
TCA intermediates from WFO	Microbial conversion	WFO	CA	1.32	[34]
	Microbial conversion (with wastewater)	WFO	CA	1.03	[45]
	Microbial conversion	Raw glycerol	KGA	0.44	[38]

^a^All conversion factors are dimensionless (referring units of mass to units of mass)

^b^Extrapolated from apple pomace silage: 1000 kg containing 345 kg NFC yield 32 kg carboxylic acids

^c^Conversion factors for idealized estimation based on prospected technological improvement

To estimate, how much of a certain product (i.e., adipic acid, carboxylic acids, or TCA cycle intermediates) can theoretically be produced from biogenic residues, the relevant fraction of all resources favorable for the respective process was subsumed and the potential product amount was calculated by applying the conversion factors. In addition to this state of the art assessment of the product potential, an idealized estimation was made by assuming increase in either availability of raw material and/or the efficiency of biotechnological conversion due to technological improvement based on [25, 32].

For better contextualization, these potential product amounts based on residues accruing in Germany were compared with actual production. Since national production is not publicly available, Germany’s share on global gross domestic product (GDP) of ca. 3% [46] was used to break down global production to national level for every product to serve as a measure for biogenic product potential.

To indicate how efficient a certain residue can be converted to a product, the utilization ratio of the raw material ${\eta }_{RM}$ is given. It is defined as

$$\eta_{RM} = {\raise0.7ex\hbox{${m_{P} }$} \!\mathord{\left/ {\vphantom {{m_{P} } {m_{RM} }}}\right.\kern-0pt} \!\lower0.7ex\hbox{${m_{RM} }$}}$$

with ${m}_{P}$ and ${m}_{RM}$ being the masses of product and raw material [in Mg], respectively. The ratio relates the biogenic product potential to the technical potential of the residues used.

## Results

### Resource matrix

The biochemical composition of biogenic residues with a technical potential > 250,000 Mg a^−1^ was analyzed in detail and arranged in a resource matrix (see Table S2). The first dimension shows the DM fractions of cellulose, hemicellulose, lignin, NFC, proteins, fats/oils, and ashes, respectively, and the second dimension considers the respective biogenic residues. The data quality score and the reference to original studies is given as additional information. The biogenic residues are sorted in descending order on their technical potential according to the DE Biomass Monitor [10, 11]. As the mass fractions of the biogenic residues are highly variable, the minimum, average, and maximum of each fraction are given where available.

The total technical potential of all 77 biogenic residues accounts to 108.8 Mio Mg a^−1^ [10, 11]. The 35 biogenic residues listed in the resource matrix have a total technical potential of 106.5 Mio Mg a^−1^; this corresponds to 97.8% of the total technical potential of all biogenic residues accruing in Germany. For 4.1 Mio Mg a^−1^ (3.7%) of those, no use as feedstock for biotechnological processes was found. The vast majority of 102.4 Mio Mg a^−1^ (94.1%) has a fundamental use as feedstock for biotechnological processes.

**Fig. 2:**
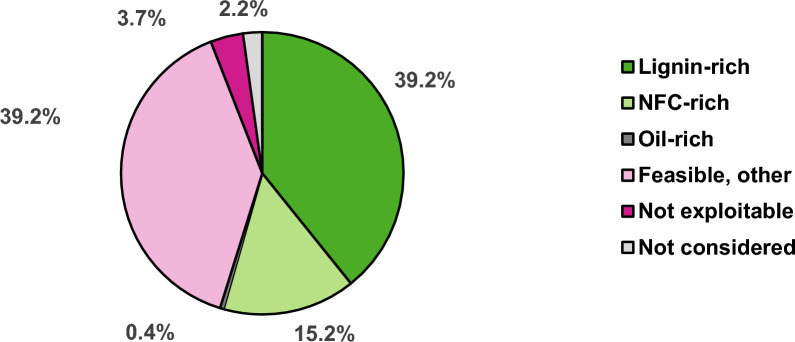
Feasibility of biogenic residues as feedstock for bioprocesses. Lignin-rich (green), NFC-rich (light green), and oil-rich (dark grey) resources collectively make up the majority of total biogenic residues. A principal feasibility to serve as feedstock for bioprocesses can be assumed for most of the other residues (rose). A minor share was found not to be exploitable (pink) or was not considered in this study due to the cutoff of 250,000 Mg a^−1^ (light grey); 100% = 108.8 Mio Mg a^−1^

According to Table 1, the data quality of 8 resources are categorized as “good”, 14 as “satisfying”, 4 as “sufficient”, 6 as “deficient” and 2 “not reliable”. Figure 2 illustrates which share of the accruing total biogenic residues can be exploited: 54.8% can be used in one of the three model processes considered, for additional 39.2%, a principal feasibility to serve as feedstock for bioprocesses can be assumed. 3.7% were found not to be exploitable using current biotechnology methods and 2.2% were not considered in this study.

Unlike existing studies on the biochemical composition of raw materials which focus on specific sectors [18] or covering only a few resources [19], this survey is broad and highly detailed and, therefore, transferable to an international context.

### Assessment of the biogenic potential of biotechnological model processes

Based on the resource matrix, the product potential of biotechnological processes when mobilizing suitable biogenic residues was estimated. To this end, (i) favorable raw materials were selected; (ii) the relevant fraction was calculated; and (iii) conversion factors from state-of-the-art literature were applied to estimate the total product quantity for each process. Since every process has specific raw material requirements, conversion specifications, and TRL, the reader is provided with a short description here.

### Polymer bricks from lignin

**Fig. 3:**
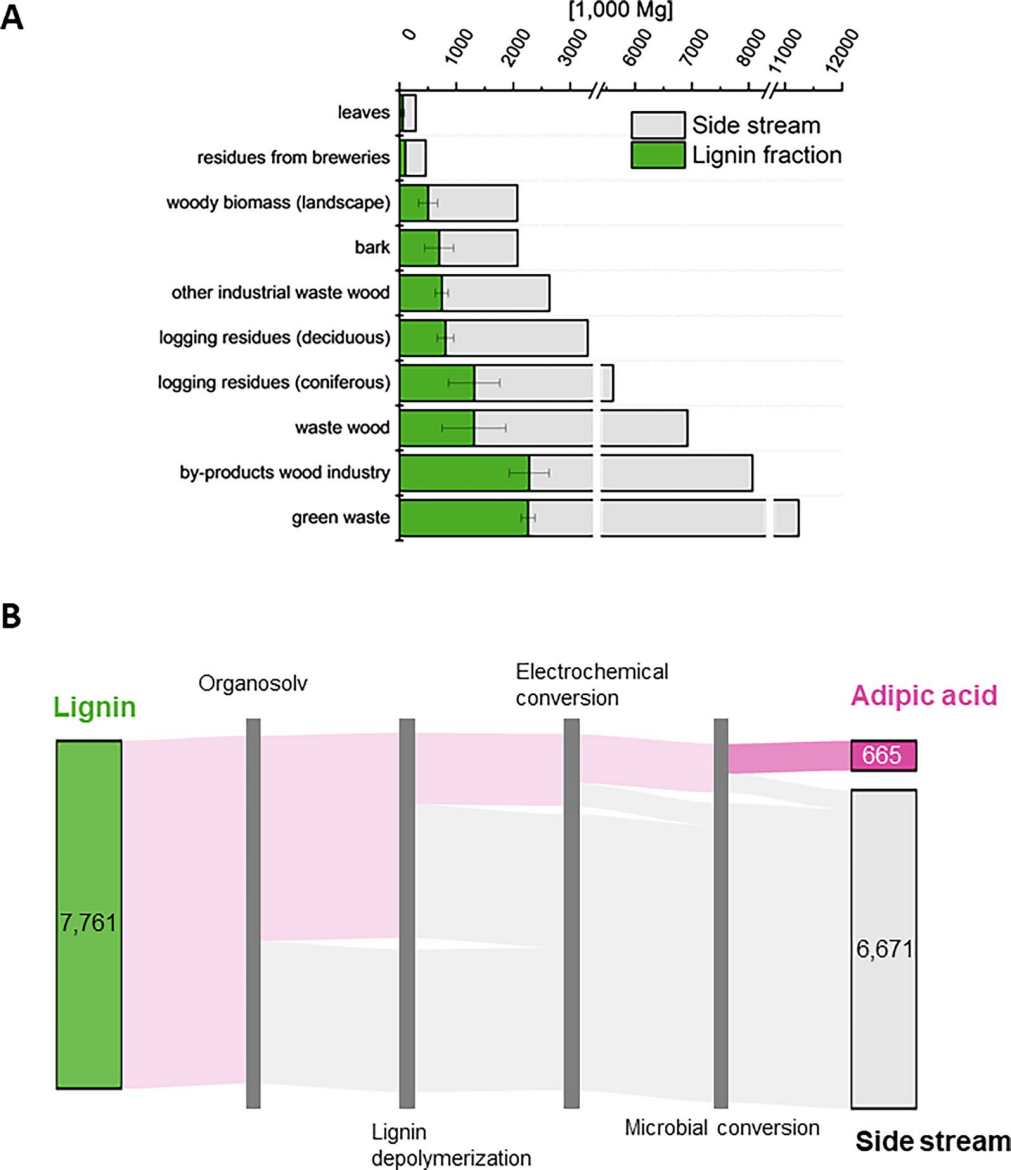
Favourable resources (**A**) and material flow (**B**) for the production of polymer brick adipic acid from lignin. **A** Ten resources sorted along their technical potential (grey bars) possess a specific lignin fraction (green bars) of ≥ 20% (DM), making them suitable feedstocks. **B** Lignin fraction needs to be separated in an organosolv process and depolymerized to be electrochemically and microbially converted to adipic acid. The sum of shares of lignin in the favorable resources (green bars in **A**) amounts to the total lignin in the material flow [green pillar in (**B**)]. A more detailed material flow chart is included in the supplementary material (Figure S2). Numbers in 1000 Mg a^−1^

This bioprocess combines electrochemical and microbial conversion to convert lignin-derived monomers to adipic acid [25]. Phenolic monomers are electrochemically hydrogenated at ambient conditions in aqueous solution before the obtained cyclic alcohols are converted to adipic acid by *Pseudomonas taiwanensis*. Phenol, syringol, guaiacol, and catechol are the predominant aromatic monomer bricks in lignin [22]. Their electrochemical hydrogenation yields intermediary cyclohexanol as well as other functionalized cyclohexanols and cyclohexane diols, which all can be microbially converted to adipic acid (unpublished data). Before, intensive pre-treatment of raw material is required: the ideally lignin-rich (lignocellulosic) biomass has to be decomposed to isolate the lignin fraction. Subsequently, the complex lignin structure needs to be depolymerized, preferably to the aromatic monomers before electrochemical conversion (Figure S1). Recently, significant efforts were put in the development of a relatively mild organosolv process using an ethanol–water mixture in combination with sulfuric acid to separate the biomass fractions while preserving their macromolecular structure [22]. Here, lignin was cleaved via base-catalyzed depolymerization. While this pretreatment implies significant loss of raw material, the mobilization of often energetically used lignin-fractions as feedstock for polymer production is visionary. Furthermore, by the here described process adipic acid is up to now only available at low titers and with low space time yield. While the combination of electrochemical and microbial conversion is a promising approach, the technology is still under development, being at TRL 2–3 today. Feedstock with high lignin content is in general favorable for the two-step process producing adipic acid (Table 3). Eight resources have lignin contents ≥ 24% (DM), namely, waste wood, by-products of wood processing industries and other industrial waste wood, residues from breweries, deciduous and coniferous logging residues, bark, and woody biomass from landscape management (Fig. 3A). Two further resources, green waste and leaves, depict a lignin content of ca. 20% (DM). Summarized, 31.2 Mio Mg a^−1^ (42.7 Mio Mg a^−1^, including green waste and leaves) of favorable resources are principally feasible for exploitation towards adipic acid production, with a total lignin fraction of 7.8 Mio Mg a^−1^ (10.1 Mio Mg a^−1^).

**Table 3:** Feasible residues for the three model processes. Based on an excerpt from the resource matrix (Table S2), residues are assigned to the process for which they can serve as feedstock and sorted by the mass fraction of dry matter of the respective biomass fraction

Model process	Resource	Description	Relevant fraction ^a^	Data quality score ^b^	References
Polymer bricks from lignin	Bark		0.34		[47–50]
	By-products of wood processing industry		0.28		[51–54]
	Other industrial waste wood		0.28		[51–55]
	Residues from breweries	Brewer’s spent grain	0.28		[56]
	Logging residues (deciduous)		0.25		[18, 57–61]
	Woody biomass from landscape management	20% conifer., 80% deciduous	0.24		[18, 19, 57–63]
	Waste wood	80% conifer., 20% deciduous [21]	0.24		[18, 19, 55, 57–63]
	Logging residues (coniferous)		0.23		[18, 19, 62, 63]
	Green waste		0.20		[64, 65]
	Leaves	Average of mixed leaves, oak, chestnut, beech	0.20		[66, 67]
Carboxylic acids from NFC	Residues from bread and bakeries production		0.65		[68, 69]
	Residues from starch production	Mainly potato pulp (80% [69])	0.53		[70, 71]
	Residues from milk processing	Mainly whey from cheese productions (99% [69])	0.52		[72, 73]
	Residues from sugar production	Average of molasse and sugar beet pulp [74]	0.40		[75–78]
	Kitchen and canteen wastes		0.38		[79–82]
	Residues from bioethanol production	Average of vinasse and sugar beet pulp [74]	0.23		[75, 76, 83]
	Cattle solid manure		0.16		[84–88]
	Stalks from roadside		0.16		[89–93]
TCA cycle intermediates from WFO	Glycerol from biodiesel production		0.95		[38]
	Oil waste		0.91		[68]

^a^Relevant fractions are lignin, NFC, or oil/glycerol depending on the model process, given in g g^−1^ (DM)

^b^ good  satisfying  sufficient  deficient not reliable, for details see Table 1

The possible multi-step production process to gain adipic acid from these lignin fractions is illustrated in Fig. 3B: based on the conversion factors for biomass decomposition [22] and lignin depolymerization [43] as well as for electrochemical and microbial conversion [25], an amount of 665,000 Mg a^−1^ adipic acid could theoretically be produced. In the context of a global annual adipic acid production of 4.5 Mio Mg, the national demand for this compound is here estimated to be around ca. 135,000 Mg a^−1^ adipic acid for Germany (see 2.4.). To meet this production with biogenic residues alone, 1.6 Mio Mg a^−1^ of lignin, which is 20.3% of the feasible lignin fractions, would suffice.

In a best case study, a more efficient lignin depolymerization (50% instead of 35% monomeric yield [44]) and, both, more efficient electrochemical and microbial conversions (product yields: 85% instead of 68%, and 99% instead of 61%, respectively) were assumed. The increased product yields for the latter two-step conversion are deduced from results of lab scale experiments, underlining the potential of this combined approach. The optimized conversion would reduce the resource demand to only 7.0% of the suitable lignin fraction to meet the national production of 135,000 Mg a^−1^ adipic acid.

### Carboxylic acids from non-fibrous carbohydrates

The Capraferm^®^ process converts feedstock containing NFC into medium-chain carboxylic acids (MCC), predominantly caproic (C_6_) and caprylic (C_8_) acid. The remaining feedstock fraction is then converted to biogas and fertilizer. Overall, the raw materials usable for Capraferm^®^ are the same feedstock that are currently used in biogas plants. The conversion is realized by anaerobic fermentation with microbial communities, which can be controlled via external parameters, such as pH and temperature [29, 33]. Biomass pre-treatment and sterile operation are dispensable, which helps to keep the process costs low. In general, the Capraferm^®^ technology can be integrated with existing biogas plants thereby reducing investments costs (Figure S1). A variation of the process uses mixotrophic microbial communities that are able to consume syngas in parallel to the organic feedstock, increasing the carboxylic acid yield, and is considered for the ideal scenario [33]. The Capraferm^®^ technology is currently at TRL 5.

**Fig. 4:**
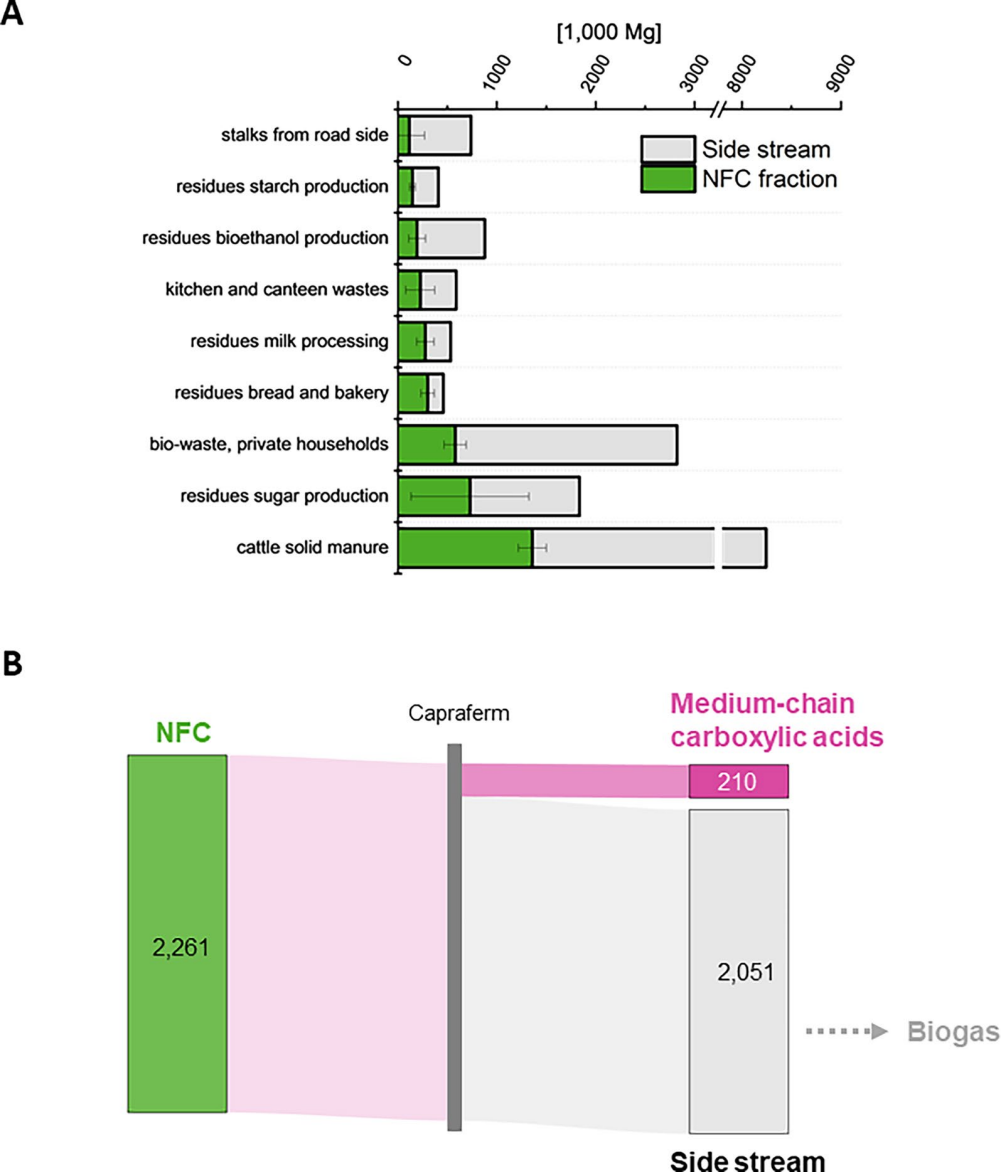
Favorable resources (**A**) and material flow (**B**) for the production of carboxylic acids from NFC. **A** Nine resources depicted with technical potential (grey bars) possess a specific NFC fraction (green bars) of ≥ 16% (DM), making them suitable feedstocks for the medium-chain carboxylic acids production. **B** Resources can directly be applied in the Capraferm® process, the side stream (fermentation residues) can be used for biogas production. The sum of shares of NFC in the favorable resources (green bars in **A**) amounts to the total NFC in the material flow (green pillar in **B**). Numbers in 1000 Mg a^−1^

Favorable resources for the Capraferm^®^ technology are characterized by a high amount of easily fermentable carbohydrates (Table 3). In particular, high shares of ethanol or lactic acid are preferred, as they are known to increase the product yield [94]. Therefore, it often is beneficial to silage raw material prior to carboxylic acid fermentation. With respect to their high share of NFC, residues from bread and bakery production, bioethanol production, starch production, milk processing, sugar production and bio-waste from private households are favorable resources (Fig. 4A). In addition, kitchen and canteen wastes, cattle solid manure, and stalks from roadside can be considered as feedstock for the Capraferm^®^ process. Taken together, the favorable raw materials account to a technical potential of 6.9 Mio Mg a^−1^ (16.6 Mio Mg a^−1^, when considering additionally the possible feedstock), with an NFC fraction of 2.7 Mio Mg a^−1^ (4.0 Mg a^−1^). From the residues from starch production only 80% of total available mass was considered, representing the share of potato pulp [21].

The Capraferm^®^ process, where these raw materials can be converted without any costly pre-treatment, could yield up to 210,000 Mg a^−1^ (368,000 Mg a^−1^) C_6_/C_8_ carboxylic acids (Fig. 4B), extrapolating yields obtained with fruit pomace silage. The corresponding carboxylic acids could be converted to up to 262,000 Mg a^−1^ (460,000 Mg a^−1^) of ingredients for lubricant formulations which is a relevant fraction of the German lubricant market, estimated to account 840,000 Mg a^−1^ in 2022 [95]. Noteworthy, when integrated in a biogas plant, biogas can be produced in parallel or downstream to the Capraferm^®^ process [32]. In the light of a global market size of 1.8 Mio Mg a^−1^ C_6_/C_8_ carboxylic acid, the assumed resource demand for Germany of about 54,000 Mg a^−1^ could be covered from biogenic residues alone, if only 25.7% of the favorable (14.7% of the possible) resources were utilized.

### TCA cycle intermediates from waste cooking fat and oil

CA can be produced from waste oils and fats and KGA from raw glycerol by means of bioconversion (Figure S1). In this example, the yeast *Y. lipolytica* is used as production host. It requires N-limitation for CA and thiamine limitation for KGA production, respectively [34, 38, 96–98]. Efficient conversion of raw glycerol with high yields has been demonstrated [38]. Likewise, feasibility of the CA production from waste cooking oil has been shown, reaching up to 145 g L^−1^ CA and a selectivity of more than 90% [34]. Intriguingly, unsterile reactor operation is in place [45]. Today, the technology poses at a TRL of 4 for KGA and 5–6 for CA.

**Fig. 5:**
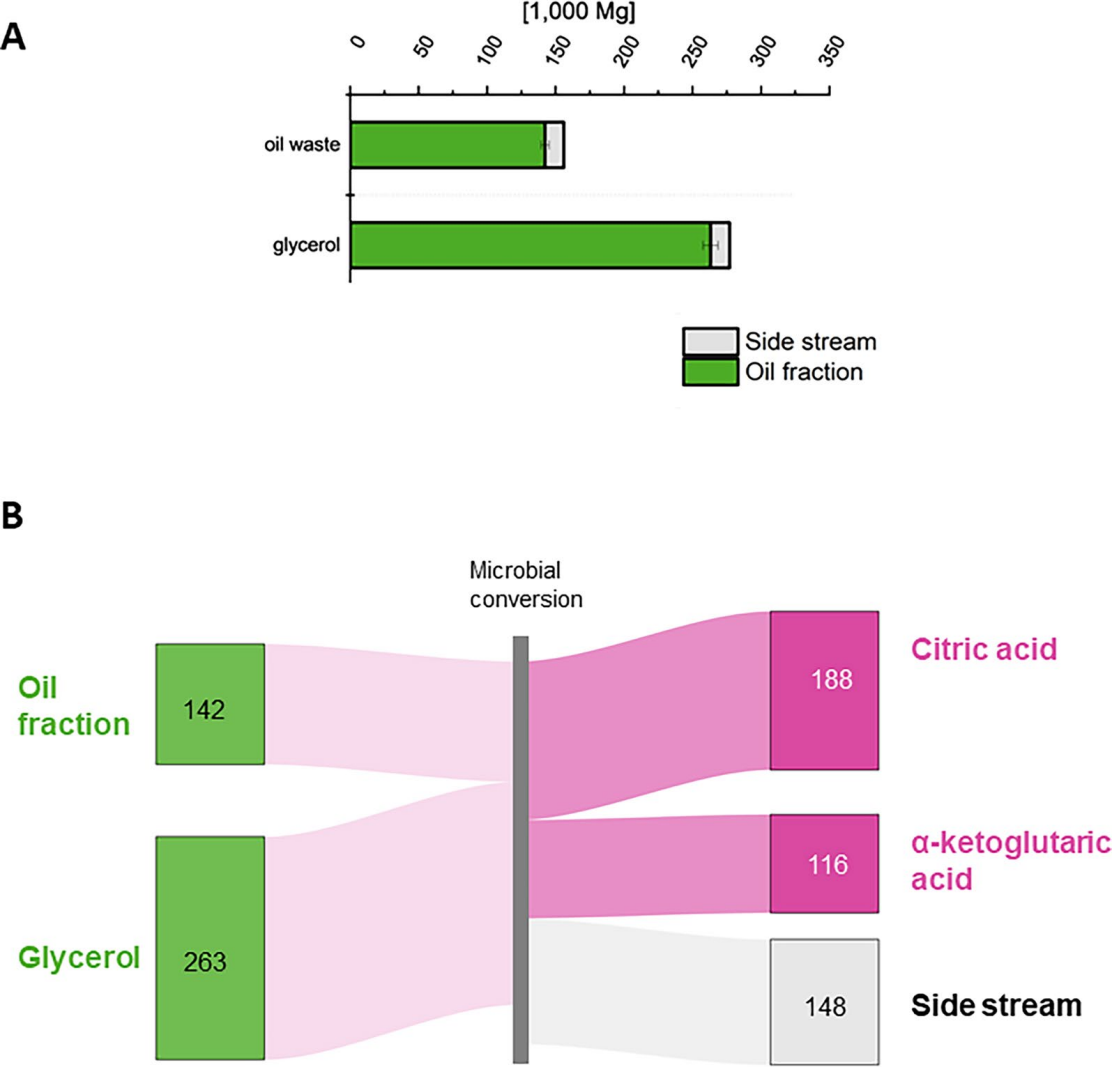
Favorable resources (**A**) and material flow (**B**) for the production of CA and KGA from WFO and glycerol. **A** Oil waste and glycerol depicted with their technical potential (grey bars) possess a specific oil/glycerol fraction (green bars) of ≥ 90% (DM), making them suitable feedstocks for TCA cycle intermediates production. **B** Resources can be microbially converted to CA and KGA. Numbers in 1000 Mg a^−1^

CA production from waste oils requires a resource with an oil content of over 90%, which limits the possibilities to oil waste (Fig. 5A). Similarly, microbial KGA production from raw glycerol demands over 90% glycerol content, making biodiesel-derived raw glycerol suitable (Table 3).

The conversion of 142,000 Mg a^−1^ oil waste in the mentioned *Y. lipolytica*-based process could yield up to 188,000 Mg a^−1^ CA (Fig. 5B), extrapolating yields from a pilot-scale study, where canteen waste oil was used as feedstock [34]. Alternatively, this process was also demonstrated using the same feedstock and fermentation wastewater as process water [45]. If the yields achieved are extrapolated, 147,000 Mg a^−1^ can be produced. For KGA production from raw glycerol, about 116,000 Mg a^−1^ KGA could be produced (Fig. 5B). To meet the resource demand for Germany of 68,000 Mg a^−1^ CA (annual production of ca. 2 Mio Mg [99]) with biogenic residues alone, 36.2% of oil waste would be required. The ${\eta }_{RM}$ for the production of TCA intermediates is the highest among the tested bioprocesses with 1.20 Mg_CA_ Mg_oil waste_^−1^ and 0.42 Mg_KGA_ Mg_glycerol_^−1^.

**Fig. 6:**
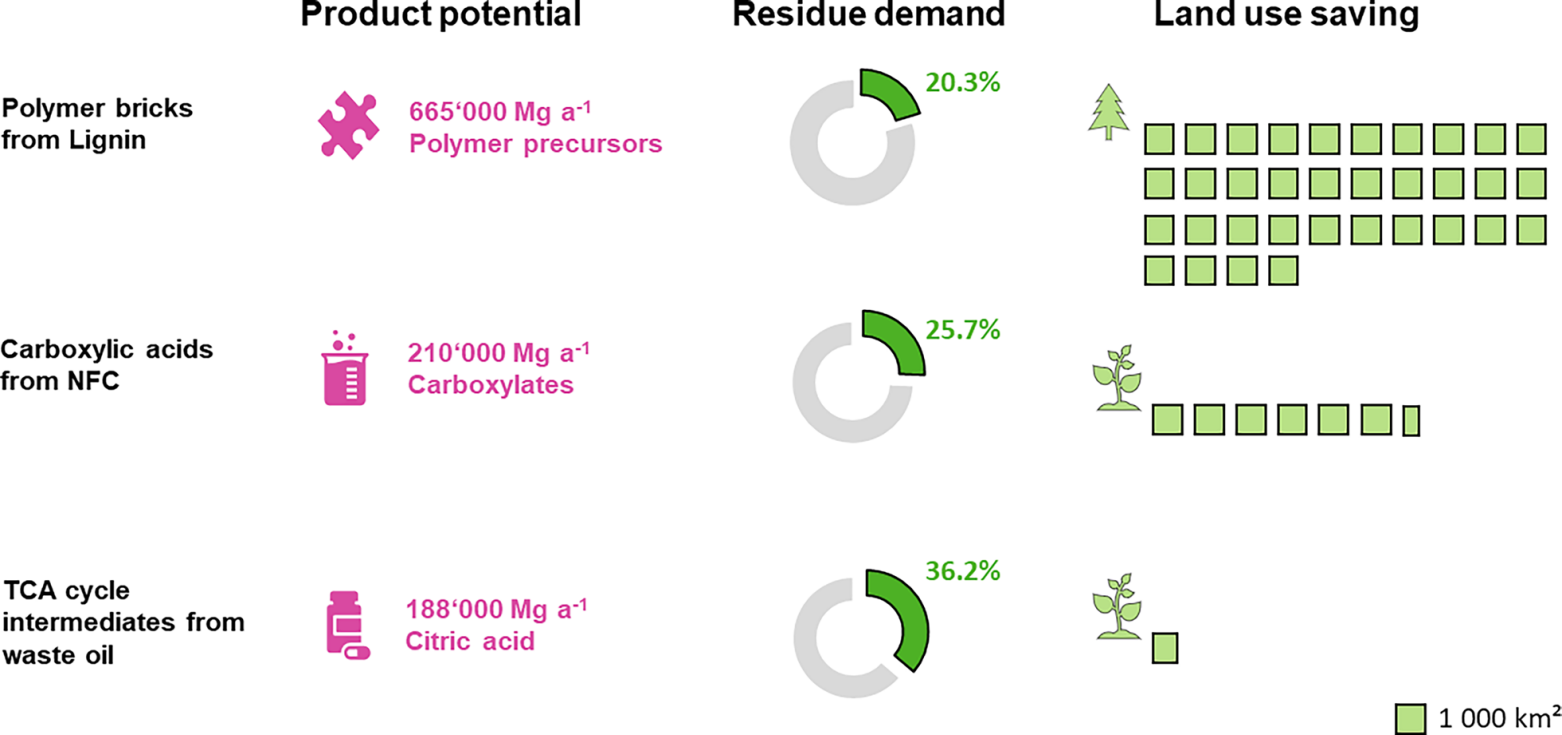
Biogenic product potential, residue demand and possible land use saving of selected model proceses. Left: possible annual volumes of specific products based on biogenic residues is given in pink. Center: green sections of the pie chart give the percentage biogenic residues that needs to be mobilized to meet national demands for respective products in Germany. Right: light green squares illustrate the potential land use saving when consequently utilizing biogenic residues. NFC, non-fibrous carbohydrates; TCA, tricarboxylic acid. For details, see main text

Table 4 summarizes the tonnages of inputs (raw material and relevant biomass fraction) and output (biogenic product potential, i.e., the potential product amount from biogenic residues) for the three model processes together with the utilization ratio ${\eta }_{RM}$. The production of the polymer brick adipic acid from lignin has the lowest ${\eta }_{RM}$ (0.021 Mg_adipic acid_ Mg_residue_^−1^_),_ but depicts the highest biogenic product potential of the three processes due to a broad resource basis. In the best-case scenario, including also resources with lignin contents between 20 and 24% (DM), namely, green waste and leaves, up to 2.5 Mio Mg a^−1^ of adipic acid could be produced from biogenic residues (Fig. 6). Similarly, the broad resource basis for the production of medium-chain carboxylic acids from NFC-rich material has a low ${\eta }_{RM}$ (0.03 Mg_carboxylic acid_ Mg_residue_^−1^), but yields 210,000 Mg a^−1^ product from ca. 6.9 Mio Mg raw material. However, the side stream from this process can still be used for biogas production. Utilizing also less favorable, but technical possible resources with NFC contents between 16 and 20% (DM), up to 210,000 Mg a^−1^ product could be reached (Fig. 6). The production of TCA cycle intermediates from WFO and raw glycerol has a very narrow feedstock basis; in this case the high ${\eta }_{RM}$ of 1.20 Mg_CA_ Mg_oil waste_^−1^ and 0.42 Mg_KGA_ Mg_Glycerol_^−1^, respectively, are key to achieve a significant biogenic product potential.

**Table 4:** Tonnages of raw material, relevant biomass fraction, and product for specific bioprocesses

Process	Raw material [Mg a^−1^]	Relevant fraction [Mg a^−1^]	Biogenic product potential [Mg a^−1^]	Utilization ratio [Mg Mg^−1^]
Polymer bricks from lignin				
Favorable resources	31.2 Mio	7.8 Mio	665,000 (2.5 Mio)^a^	0.021 (0.059)^a^
+ possible resources^b^	42.7 Mio	10.1 Mio		
Medium-chain carboxylic acids from NFC				
Favorable resources	6.9 Mio	2.7 Mio	210,000	0.030
+ possible resources^b^	16.6 Mio	4.0 Mio	368,000	
TCA cycle intermediates from WFO				
Waste oil	156,000	142,000	188,000 [CA]	1.20
Glycerol	277,000	263,000	116,000 [KGA]	0.42

^a^Best case scenario assuming expectable improvements in lignin depolymerization, electrochemical and microbial conversion (see text)

^b^Possible resources are green waste and leaves (lignin content between 20% and 24% (DM) and cattle solid manure and stalks from roadside (NFC content between 16% and 20% (DM), respectively. Mass values on these lines reflect the total sum when using both favorable and possible resources

## Discussion

We analyzed the potential of biogenic residues accruing in Germany as feedstocks for biotechnological production processes. 35 from 77 residues listed in the DE biomass monitor were screened, covering a total amount of 97.8% of the listed technical potential [10]. Three processes at different TRLs, using lignin-rich, NFC-rich, and oil-rich resources, respectively, served as model processes to estimate the theoretical production potential based on biogenic residues (Fig. 6).

While the potentials of biogenic residues per capita can be expected to be somewhat comparable on a global scale [100], it is obvious that different situations are faced in different countries. Therefore, we discuss the effects on national level in the following. The here presented numbers provide evidence that biogenic residues can contribute significantly to net zero land solutions. Remarkably, for the three model processes alone, a total of 59.7 Mio Mg a^−1^ (54.8%) of biogenic residues were found to be suitable raw materials. Thus, we can deduce a generally high biogenic product potential. These findings shall provide guidance for net zero land (or circular bioeconomy) when comparing possible product amounts to the respective production.

Depending on the bioprocess and the type of product, only 20–30% of the possible raw material needs to be mobilized on the national level for the respective bioprocess to cover demands of specific products based on biogenic residues alone. Nevertheless, the discussed model processes are expected to challenge fierce competition for these residues in a future circular bioeconomy. In this context, basing the material flows on the technical potential might be pragmatic. This, however, veils the truth that existing conventional processes already compete for the residues of interest, reducing the available theoretical potential. Yet, existing processes often use residues for energy generation only. In line with a circular bioeconomy, the bioprocesses should use the naturally grown (chemical) structure for tailor-made products, such as biopolymers [25, 39] or pharmaceuticals [36, 37, 39]. This is highly advantageous from both the economic and ecologic perspective, as production comes along with higher added value and material flows can be tapped that are currently unused.

From a systematic perspective, the three investigated model processes are intriguing, as they pose specific challenges, enabling differential conclusions for the transition towards a circular bioeconomy: in the first example, the value-added polymer precursor adipic acid is produced from the lignin fraction (of mostly wood-derived residues) by means of biochemical, electrochemical, and microbial conversion. Due to its complex structure and, thus, breakdown, lignin is often preferably energetically used. Yet, the interest in making material use of lignin is increasing and targeted decomposition processes are developed [22, 43]. Despite the attractive product, the low η_RM_ is insufficient for large-scale production. Considering the currently still low TRL, the conversion efficiencies can be expected to improve, but it is already obvious that adipic acid cannot be the only product from the respective residues. A composite approach is required to mobilize the respective raw material to its full potential. In the light of circular bioeconomy ambitions, the efficient usage of resources and residues, as well as creating valuable by-products and joint products will be key [101]. For (material) usage of lignocellulosic biomass, the development of decomposition methods enabling a separate valorization of each fraction, e.g., aromatic monomers, is very promising [22].

The production of medium-chain carboxylic acids from NFC-rich residues with the Capraferm^®^ technology can rather simply be combined with existing processes. Besides having a relatively high TRL, anaerobic fermentation to carboxylic acids uses only a small share of the feedstock and can be done prior to its use in conventional anaerobic digestion plants. Compared to biogas, the typical product of such plants, medium-chain carboxylic acids have high market prices [33], justifying the comparably low utilization ratio ${\eta }_{RM}$ of 0.03 Mg_carboxylic acid_ Mg_residue_. However, two main challenges can be anticipated: first, shifting the present paradigm of the production of a single energy carrier (i.e., methane) to the production of specialty chemicals raises the issue of both technology acceptance by the biogas plant operator and legal framework and security. Second, the question of target markets needs to be addressed: medium-chain carboxylic acids have a wide range of applications, such as nutrition, fragrances, flavors, food ingredients, and lubricants [30], albeit with different specifications and requirements. Identifying the most promising target product, will likely determine the future of this process concept. The low ${\eta }_{RM}$ of the lignin- and NFC-utilizing processes reflects that only a small share of the inputs ends up in the product fraction. The majority of the used residue is still a residue after the process, which could be used for other process lines. This underlines the necessity to develop biotechnology processes in networks from an early stage on to successfully establish an integrated circular bioeconomy. Furthermore, as biogenic resources and residues accrue decentralized, residues-based biotechnological production requires decentralized design to keep transportation efforts low.

The production of TCA cycle intermediates from vegetable oil residues or raw glycerol can be conducted with a compellingly efficient resource use (${\eta }_{RM}$ of 1.20 Mg_CA_ Mg_oil waste_^−1^ and 0.42 Mg_KGA_ Mg_glycerol_^−1^, respectively). One bottleneck for increasing the production from residues is a limitation by the resource itself. In this regard, the separation of oil waste in private households could help to increase the feedstock [102]. Besides, depending on the target market, legal restrictions come into play. In principle, CA and KGA are versatile educts for the use as an additive in food and beverages, and beyond that for pharmaceutical synthesis and medical application [36, 37, 39], but the residue-based production often legally prohibits their use in the pharmaceutical and arguably in the food sector as residues are still considered as a waste rather than a resource. Here, the legal framework needs to be adjusted to allow implementing products in a target market based on their quality rather than on the origin of their feedstock. On the other hand, there are a number of interesting applications in the technical field such as cleaners, decalcifiers, chelating agents, or co-polymers that allow the use of CA and KGA obtained from waste products [36, 37]. Intriguingly, this bioprocess is an excellent example for early stage sustainability assessment being a useful tool to control and reduce environmental impact as early as possible in process development [34].

In an envisioned bioeconomy the production of multiple goods and services is expected to compete for arable land. Since the early 2000s, the global biofuels sector has expanded significantly, fueling the debate, if available arable land should be used for food, fodder, fuel, or fiber [103]. While bioenergy will play a central role in bioeconomy transition, environmental and socio-economic implications need to be considered [104, 105]. Utilizing biogenic residues is an important measure to reduce land use, and thereby respective conflicts [106]. Put into numbers, producing 650,000 Mg of adipic acid based on residues instead of, e.g., trunk wood has the potential to save more than 3 Mio ha woodland (assuming a yield of 10 Mg ha^−1^ [107], see Fig. 6). Likewise, the residue-based production of 210,000–368,000 Mg medium-chain carboxylic acids and 188.000 Mg of CA could save could save 0.70–1.2 Mio ha arable land that would otherwise be required for the production of corn silage and rapeseed oil (assumption: 40 Mg ha^−1^ crop yield with a DM of 35% for corn silage [108], and 40 Mg ha^−1^ with an oil content of 44.6% for rapeseed oil [109]). While these numbers are no direct comparison to an existing status quo, they emphasize the tremendous potential of mobilizing biogenic residues as feedstock for production processes.

Furthermore, research and development should specifically address the exploitation of residues that cannot be exploited with existing technologies. For lignin this is exemplified here with a process resembling an electro-biorefinery [110]. But also other purely electrochemical approaches exploiting lignin-rich black water to gain vanillin need to be considered [111].

The available resource potential database (DE-Biomass Monitor [13]) for Germany lists data for 77 biogenic by-products, residues and waste with reference years 2010–2020. The resource information is differentiated into various levels of potential, but mainly focuses on energy conversion relevant parameters, such as water content or calorific values. The potential information can up to now only be contextualized in terms of its relevance in various bioenergy products for the transport sector. Future research needs to address possible demands of biogenic by-products, residues and waste across all sectors of the bioeconomy. In case of the chemical sector this requires an implementation of the chemical compositions of the biomasses, describing possible bio-based products (e.g., base chemicals and polymers) and the resulting relevance (e.g., market share in the chemical sector).

In the context of policy strategies, our findings are in good agreement with the claim to increase the use of biogenic residues communicated, e.g., in the German national bioeconomy strategy [112]. While this study cannot be universally valid for every endeavour on how to utilize biogenic residues as feedstocks, it is an important blueprint on how to approach selected processes. By investigating three specific processes, we are able to infer general conclusions being valid independently from the region: one key learning is that it is not feasible to mobilize a certain residue for a single product alone, but that there is the urgent need for integrating several process lines to utilize existing residues to their full potential. To achieve this, however, the availability and accessibility of (detailed) information on biochemical composition is required to simplify ex ante assessment how and to which biogenic residues can be utilized. When reliable resource data with high quality is made available, this will improve decision-making opportunities with regard to the utilization of residues as resources for the transformation to a more circular bioeconomy.

## Conclusion and outlook

This study provides a cross-sectoral resource matrix comprising detailed biochemical information for specific biogenic residues, which is expected to be transferable into an international context. The biochemical composition of most biogenic residues in Germany indicates that they are generally suitable as feedstock for bioprocesses. The three model processes investigated were shown to have the potential to produce relevant tonnages of medium-chain carboxylic acids, the nylon precursor adipic acid, and platform chemicals such as CA and KGA exclusively from biogenic residues. However, the often-observed low utilization ratio of the raw material calls for cluster approaches to ensure efficient cross-linking of multiple production steps combined with considered decomposition of complex biomass to exploit biogenic residues to their full potential. Furthermore, implementation of existing technologies needs to be accelerated by overcoming acceptance and legal limitations, such as the production of, e.g., pharmaceuticals from residues. In some cases, waste management needs to be optimized to increase the availability of valuable residues, such as used cooking oil. Finally, making reliable and detailed data on the biochemical composition available to will be of key importance to support transition to a circular bioeconomy.

While the focus on tonnages and production capacities is a first step towards realizing circular bioeconomy, many more questions need to be addressed: besides the material and product estimation, a detailed energetic assessment is required, respecting the fact that most of the biogenic residues are still energetically used. Furthermore, the assessment of environmental impacts and carbon footprints are expected to complement this study to answer the question of the most sustainable resource use. For integration into existing markets, an economic assessment is required to determine which raw material can be used for the production of which commodity, fine chemical and consumable. It is essential to conduct and consider these assessments as early as possible in (bio)process development to enable transformation of today’s linear production into the circular bioeconomy.

## Supplementary Information

Additional file 1. The supporting information comprising a glossary and the extended resource matrix are available online

## Data Availability

No data sets were generated or analysed during the current study.
