# Supplementary material for: Towards net zero land biotechnology: an assessment of biogenic feedstock potential for selected bioprocesses in Germany
Source: Biotechnol Biofuels Bioprod. 2025 Jul 9;18:69. doi: 10.1186/s13068-025-02673-y (PMC12243329; doi:10.1186/s13068-025-02673-y)
Supplement: Supplementary file 1 — Additional file 1. The supporting information comprising a glossary and the extended resource matrix are available online [file 13068_2025_2673_MOESM1_ESM.docx]

**Towards net zero land biotechnology – an assessment of biogenic feedstock potential for selected bioprocesses in Germany**

**Supporting Information**

**Authors**

Adrian Tüllinghoff ^a,^ * [adrian.tuellinghoff@ufz.de](mailto:adrian.tuellinghoff@ufz.de), Heike Sträuber ^a^ [heike.straeuber@ufz.de](mailto:heike.straeuber@ufz.de), Flávio César Freire Baleeiro ^a^ [flavio.baleeiro@ufz.de](mailto:flavio.baleeiro@ufz.de), Andreas Aurich ^b^ [andreas.aurich@ufz.de](mailto:andreas.aurich@ufz.de), Micjel Chávez Morejón ^a^ [micjel.chavez-morejon@ufz.de](mailto:micjel.chavez-morejon@ufz.de), Kathleen Meisel ^c^ [Kathleen.Meisel@dbfz.de](mailto:Kathleen.Meisel@dbfz.de), Karl-Friedrich Cyffka ^c^ [Karl-Friedrich.Cyffka@dbfz.de](mailto:Karl-Friedrich.Cyffka@dbfz.de), Falk Harnisch ^a^ [falk.harnisch@ufz.de](mailto:falk.harnisch@ufz.de), Katja Bühler ^a^ [katja.buehler@ufz.de](mailto:katja.buehler@ufz.de), Daniela Thrän ^d^ [daniela.thraen@ufz.de](mailto:daniela.thraen@ufz.de)

**Affiliation**

^a^ Department of Microbial Biotechnology, Helmholtz-Center for Environmental Research - UFZ Leipzig, Germany

^b^ Department of Systemic Environmental Biotechnology, Helmholtz-Center for Environmental Research - UFZ Leipzig, Germany

^c^ DBFZ-Deutsches Biomasseforschungszentrum gGmbH, Leipzig, Germany

^d^ Department of Bioenergy, Helmholtz-Center for Environmental Research - UFZ Leipzig, Germany

**Glossary**

| **Term** | **Description** | **Reference** |
| --- | --- | --- |
| Biogenic residue | Biogenic by-products or biogenic wastes | [4] |
| Theoretical potential | Maximum amount of biomass which can be considered theoretically available within biophysical limits | [15] |
| Technical potential | The share of the theoretical potential, which is available using established technologies, considering spatial restrictions as well as non-technical constraints | [15] |
| Feedstock potential | The feasibility of a residue to serve as feedstock for a specific bioprocess | This study |
| Biogenic product potential | The amount of product of a bioprocess that could be produced based on a (or all) residues | This study |
| Utilization ratio of raw material | The ratio of the product mass and the mass of raw material | This study |

**Additional information to the methodology described in 2.2.**

To compile detailed and reliable data on the biochemical composition of each resource a comprehensive research was performed including biomass databases, like Phyllis [113], reports [92, 114] and data sheets for specific residues, compiled by the DBFZ (German biomass research center) [65, 115, 116]. The main source were peer-reviewed articles being screened in a search engine based literature research. This was conducted using google scholar (latest access 2024-06-17) by combining the name of the respective residue (e.g. “green waste” or “wheat straw”) with “biochemical composition”, “biochemical structure”, or “biomass composition” as key word. The criteria to accept or reject resources were the recency, the quality of analysis and the measuring scope.

Table S2 lists the resources that were considered for the literature research, as well as examples for rejected resources.

Table S1: **List of resources being accepted or rejected in the literature research.**

| **Accepted resources** | **Author / Year** | **Reference** | **Author / Year** | **Reference** |
| --- | --- | --- | --- | --- |
|  | Adamovic et al., 1998 | [117] | Li et al., 2009 | [84] |
|  | Antal et al., 2000 | [18] | Li et al., 2015 | [88] |
|  | Bari et al., 2014 | [61] | Liu et al., 2023 | [64] |
|  | Bledzki et al., 2010 | [118] | Lynd et al., 1999 | [119] |
|  | Boadu et al., 2023 | [54] | Malakahmad et al., 2011 | [79] |
|  | Bozkurt et al., 2004 | [120] | Mayer & Hildebrandt, 1997 | [70] |
|  | Čabalova et al., 2021 | [62] | Montane et al., 1998 | [121] |
|  | Cardoso et al., 2009 | [122] | Naik et al., 2010 | [19] |
|  | Cherney et al., 1988 | [123] | Nigam, 2001 | [124] |
|  | Choi et al., 2021 | [125] | Ong et al., 2000 | [126] |
|  | Concha Olmos & Zúῆiga Hansen, 2012 | [75] | Otto et al., 2012 | [38] |
|  | Cortez et al., 2020 | [83] | Pinkowska et al, 2019 | [76] |
|  | DBFZ, 2023a | [116] | Rusanen et al., 2019 | [52] |
|  | DBFZ, 2023b | [65] | Ruxanda et al., 2008 | [60] |
|  | DBFZ, 2023c | [115] | Sander, 1997 | [127] |
|  | Filipova et al., 2012 | [63] | Schmidt et al., 2019 | [114] |
|  | Fourty et al., 1996 | [67] | Shen et al., 2019 | [128] |
|  | Fradinho et al., 2002 | [48] | Shen et al., 2019 | [85] |
|  | Frankó et al., 2015 | [47] | Singh et al., 1996 | [77] |
|  | Gaida et al., 2013 | [69] | Sjölin et al., 2019 | [78] |
|  | Gárdenas-Gutiérrez et al., 2018 | [59] | Slopiecka et al., 2022 | [68] |
|  | Hilgert et al., 2023 | [87] | Stiller et al., 1996 | [51] |
|  | Jin et al., 2012 | [50] | Tang et al., 2008 | [80] |
|  | Kangas et al., 2019 | [53] | Thrän, 2024 | [4] |
|  | Klingspohn et al., 1993 | [71] | Verhe et al., 2022 | [129] |
|  | Krenz & Pleissner, 2024 | [91] | Viretto et al., 2021 | [93] |
|  | Laskowska & Boruszewski, 2018 | [58] | Waliszewska et al., 2018 | [49] |
|  | Le Floch et al., 2015 | [57] | Waliszewska et al., 2021 | [90] |
|  | Lee, 1997 | [130] | Zhao et al., 2018 | [86] |
|  | Leming & Lember, 2005 | [131] | Zhou et al., 2016 | [132] |
| **Rejected resources** | **Author year** | **Reference** | **Reason for rejection** |  |
|  | Li et al., 2019 | [133] | Insufficient information on dataset |  |
|  | Smith et al., 2009 | [134] | Insufficient information on dataset |  |
|  | Gandam | [135] | Insufficient information on methodology | |
|  | Kiritsakis | [136] | Insufficient information on methodology | |
|  | Nazifa, et al., 2021 | [137] | Insufficient information on dataset |  |

Table S2: **Biochemical composition of biogenic residues and wastes accruing in Germany arranged in a resource matrix.** Mass fractions of cellulose, hemicellulose, lignin, NFC, proteins, fats/oil, and ashes are given in (w/w) of dry matter. Grey-shadowed resources were not found to be feasible feedstock. For details on the data quality score, see Table 1 (green = good, light green = satisfying, white = sufficient, rose = deficient, pink = not reliable). If available, minimum (MIN) and maximum (MAX) data are given in addition to averaged data (MEAN). Empty spaces: no data available.

| **Resource** | **Description** | **value** | **Cellulose** | **Hemicellulose** | **Lignin** | **NFC** | **Proteins** | **Fats/oil** | **Ash** | **Data quality ^a^** | **References** |
| --- | --- | --- | --- | --- | --- | --- | --- | --- | --- | --- | --- |
| Waste paper | Newsprint | MIN | 0.56 | 0.10 | 0.10 |  | 0.01 |  | 0.04 |  | [129, 130, 138] |
|  |  | MEAN | 0.60 | 0.15 | 0.15 |  | 0.01 |  | 0.10 |  |  |
|  |  | MAX | 0.64 | 0.22 | 0.21 |  | 0.01 |  | 0.15 |  |  |
| Waste paper | Mixed waste | MIN | 0.57 | 0.06 | 0.01 |  | 0.00 |  | 0.13 |  | [138] |
|  |  | MEAN | 0.59 | 0.09 | 0.07 |  | 0.01 |  | 0.18 |  |  |
|  |  | MAX | 0.60 | 0.12 | 0.13 |  | 0.01 |  | 0.23 |  |  |
| Green waste |  | MIN | 0.35 | 0.19 | 0.19 |  | 0.01 |  | 0.04 |  | [64, 65] |
|  |  | MEAN | 0.40 | 0.19 | 0.20 |  | 0.01 |  | 0.09 |  |  |
|  |  | MAX | 0.45 | 0.20 | 0.21 |  | 0.01 |  | 0.13 |  |  |
| Cereal straw, wheat | accounting for 51% of the crop area in Germany [139] | MIN | 0.34 | 0.24 | 0.08 | 0.01 | 0.03 |  | 0.07 |  | [19, 77, 117, 119, 121, 124, 127, 130] |
|  |  | MEAN | 0.40 | 0.28 | 0.16 | 0.07 | 0.03 |  | 0.09 |  |  |
|  |  | MAX | 0.46 | 0.31 | 0.23 | 0.13 | 0.03 |  | 0.10 |  |  |
| Cereal straw, other | barley, oat, rye | MIN | 0.29 | 0.21 | 0.05 |  |  |  | 0.06 |  | [19, 86, 123, 127] |
|  |  | MEAN | 0.38 | 0.25 | 0.16 |  |  |  | 0.10 |  |  |
|  |  | MAX | 0.51 | 0.30 | 0.23 |  |  |  | 0.14 |  |  |
| Cattle slurry |  | MIN | 0.24 | 0.04 | 0.07 |  | 0.04 |  | 0.24 |  | [115, 126] |
|  |  | MEAN | 0.26 | 0.16 | 0.12 |  | 0.04 |  | 0.24 |  |  |
|  |  | MAX | 0.28 | 0.29 | 0.17 |  | 0.04 |  | 0.24 |  |  |
| By-products of wood processing industry |  | MIN | 0.40 | 0.17 | 0.25 |  | 0.02 |  | 0.00 |  | [51-54] |
|  |  | MEAN | 0.45 | 0.22 | 0.28 |  | 0.02 |  | 0.00 |  |  |
|  |  | MAX | 0.48 | 0.26 | 0.33 |  | 0.02 |  | 0.01 |  |  |
| Waste wood | Assuming 80% coniferous, 20% deciduous [21] ^b^ | MIN | 0.32 | 0.18 | 0.12 | 0.03 | 0.00 | 0.00 | 0.01 |  | [18, 19, 55, 57-63] |
|  |  | MEAN | 0.42 | 0.27 | 0.24 | 0.06 | 0.00 | 0.00 | 0.03 |  |  |
|  |  | MAX | 0.55 | 0.35 | 0.28 | 0.14 | 0.00 | 0.00 | 0.03 |  |  |
| Residues from oil mills | Rapeseed press cake/ press meal ^c^ | MIN | 0.11 | 0.03 | 0.09 |  | 0.29 | 0.02 | 0.06 |  | [131, 132] |
|  |  | MEAN | 0.13 | 0.15 | 0.14 |  | 0.37 | 0.05 | 0.08 |  |  |
|  |  | MAX | 0.22 | 0.22 | 0.16 |  | 0.41 | 0.23 | 0.08 |  |  |
| Cattle solid manure |  | MIN | 0.18 | 0.13 | 0.08 | 0.15 | 0.06 | 0.02 |  |  | [84-88] |
|  |  | MEAN | 0.21 | 0.20 | 0.11 | 0.16 | 0.15 | 0.03 |  |  |  |
|  |  | MAX | 0.24 | 0.30 | 0.18 | 0.18 | 0.22 | 0.03 |  |  |  |
| Logging residues (coniferous) |  | MIN | 0.39 | 0.18 | 0.12 | 0.03 |  |  | 0.03 |  | [18, 19, 62, 63] |
|  |  | MEAN | 0.42 | 0.27 | 0.23 | 0.07 |  |  | 0.03 |  |  |
|  |  | MAX | 0.49 | 0.35 | 0.28 | 0.14 |  |  | 0.03 |  |  |
| Logging residues (deciduous) |  | MIN | 0.32 | 0.19 | 0.19 |  |  |  | 0.01 |  | [18, 57-61] |
|  |  | MEAN | 0.41 | 0.25 | 0.25 |  |  |  | 0.01 |  |  |
|  |  | MAX | 0.55 | 0.34 | 0.28 |  |  |  | 0.01 |  |  |
| Bio-waste from private households | | MEAN | 0,22 |  | 0.16 | 0.21 | 0.21 | 0.11 | 0.30 |  | [68, 114, 116] |
| Biogenic share of waste water | |  |  |  |  |  |  |  |  |  |  |
| Other industrial waste wood | similar to by-products of wood processing industry [55] | MIN | 0.40 | 0.17 | 0.25 |  | 0.02 |  | 0.00 |  | [51-55] |
|  |  | MEAN | 0.45 | 0.22 | 0.28 |  | 0.02 |  | 0.00 |  |  |
|  |  | MAX | 0.48 | 0.26 | 0.33 |  | 0.02 |  | 0.01 |  |  |
| Bark |  | MIN | 0.24 | 0.15 | 0.20 |  |  |  | 0.00 |  | [47-50] |
|  |  | MEAN | 0.32 | 0.20 | 0.34 |  |  |  | 0.02 |  |  |
|  |  | MAX | 0.44 | 0.29 | 0.44 |  |  |  | 0.03 |  |  |
| Woody biomass from landscape management | Assuming 20% coniferous, 80% deciduous | MIN | 0.32 | 0.18 | 0.12 | 0.03 | 0.00 | 0.00 | 0.01 |  | [18, 19, 57-63] |
|  |  | MEAN | 0.41 | 0.26 | 0.24 | 0.01 | 0.00 | 0.00 | 0.01 |  |  |
|  |  | MAX | 0.55 | 0.35 | 0.28 | 0.14 | 0.00 | 0.00 | 0.03 |  |  |
| Residues from sugar production | Weighted average of molasses and sugar beet pulp [140] | MIN | 0.00 | 0.00 | 0.00 | 0.10 | 0.09 | 0.00 | 0.00 |  | [75-78] |
|  |  | MEAN | 0.12 | 0.23 | 0.04 | 0.40 | 0.10 | 0.06 | 0.11 |  |  |
|  |  | MAX | 0.23 | 0.45 | 0.09 | 0.75 | 0.16 | 0.09 | 0.24 |  |  |
| Residues from cereal processing | | MEAN | 0,36 | 0.18 | 0.16 | 0.09 | 0.06 | 0.05 |  |  | [118] |
| Residues from meat processing | Weighted average of pig, poultry, and cow share, Germany [69] | MIN | 0.02 |  |  |  | 0.25 | 0.13 | 0.19 |  | [120, 125] |
|  |  | MEAN | 0.02 |  |  |  | 0.47 | 0.22 | 0.30 |  |  |
|  |  | MAX | 0.03 |  |  |  | 0.57 | 0.56 | 0.49 |  |  |
| Horse solid manure |  | MEAN | 0.38 | 0.34 | 0.20 |  |  |  | 0.11 |  | [51] |
| Pig slurry |  | MIN | 0.12 | 0.17 | 0.02 |  | 0.03 |  | 0.07 |  | [85, 88, 128] |
|  |  | MEAN | 0.17 | 0.20 | 0.06 |  | 0.03 |  | 0.07 |  |  |
|  |  | MAX | 0.24 | 0.22 | 0.08 |  | 0.03 |  | 0.07 |  |  |
| Residues from bioethanol production | Weighted average of vinasse and sugar beet pulp [74] | MIN | 0.00 | 0.00 | 0.00 | 0.13 | 0.09 | 0.00 | 0.00 |  | [75, 76, 83] |
|  |  | MEAN | 0.08 | 0.15 | 0.03 | 0.23 | 0.11 | 0.04 | 0.10 |  |  |
|  |  | MAX | 0.23 | 0.45 | 0.09 | 0.32 | 0.16 | 0.09 | 0.24 |  |  |
| Sewage sludge from public wastewater treatment plants | |  |  |  |  |  |  |  |  |  |  |
| Stalks from roadside ^d^ |  | MIN | 0.21 | 0.00 | 0.05 | 0.00 | 0.06 | 0.00 | 0.02 |  | [89-93] |
|  |  | MEAN | 0.34 | 0.24 | 0.19 | 0.15 | 0.11 | 0.01 | 0.09 |  |  |
|  |  | MAX | 0.44 | 0.34 | 0.30 | 0.42 | 0.17 | 0.02 | 0.25 |  |  |
| Chicken solid manure |  | MIN | 0.04 | 0.12 | 0.02 | 0.08 | 0.05 |  | 0.22 |  | [85, 88, 141] |
|  |  | MEAN | 0.13 | 0.20 | 0.02 | 0.13 | 0.10 |  | 0.22 |  |  |
|  |  | MAX | 0.20 | 0.24 | 0.03 | 0.17 | 0.16 |  | 0.22 |  |  |
| Kitchen and canteen wastes |  | MIN | 0.02 | 0.08 | 0.01 | 0.10 |  |  | 0.06 |  | [79-82] |
|  |  | MEAN | 0.15 | 0.09 | 0.09 | 0.38 |  |  | 0.12 |  |  |
|  |  | MAX | 0.23 | 0.10 | 0.17 | 0.60 |  |  | 0.19 |  |  |
| Residues from milk processing | Mainly whey from cheese productions (99% [69]) | MIN |  |  |  | 0.41 | 0.12 |  | 0.07 |  | [72, 73] |
|  |  | MEAN |  |  |  | 0.52 | 0.42 |  | 0.08 |  |  |
|  |  | MAX |  |  |  | 0.74 | 0.58 |  | 0.10 |  |  |
| Residues from breweries | Mainly brewer’s spent grain (80% [69]) | MIN |  |  |  |  |  |  |  |  | [56] |
|  |  | MEAN | 0.17 | 0.28 | 0.28 |  | 0.15 | 0.10 | 0.24 |  |  |
|  |  | MAX |  |  |  |  |  |  |  |  |  |
| Stalks from landscape management | Similar to stalks from roadside ^d^ | MIN | 0.21 |  | 0.05 |  | 0.06 |  | 0.02 |  | [89-93] |
|  |  | MEAN | 0.34 | 0.24 | 0.19 | 0.15 | 0.11 | 0.01 | 0.09 |  |  |
|  |  | MAX | 0.44 | 0.34 | 0.30 | 0.42 | 0.17 | 0.02 | 0.25 |  |  |
| Residues from bread and bakery production |  | MIN | 0.05 |  |  | 0.51 | 0.08 | 0.01 | 0.05 |  | [68, 69] |
|  |  | MEAN | 0.08 |  |  | 0.65 | 0.10 | 0.03 | 0.08 |  |  |
|  |  | MAX | 0.10 |  |  | 0.81 | 0.13 | 0.06 | 0.10 |  |  |
| Residues from starch production | Mainly potato pulp (80% [69]) | MIN | 0.17 | 0.10 |  | 0.45 | 0.03 |  | 0.03 |  | [70, 71] |
|  |  | MEAN | 0.21 | 0.13 |  | 0.53 | 0.04 |  | 0.04 |  |  |
|  |  | MAX | 0.25 | 0.15 |  | 0.60 | 0.05 |  | 0.05 |  |  |
| Biogenic share of old textiles |  |  |  |  |  |  |  |  |  |  |  |
| Leaves | Average of mixed leaves [67, 142], oak, chestnut, beech [66] | MIN | 0.19 | 0.14 | 0.09 | 0.05 |  |  | 0.06 |  | [66, 67] |
|  |  | MEAN | 0.29 | 0.26 | 0.20 | 0.13 |  |  | 0.06 |  |  |
|  |  | MAX | 0.34 | 0.35 | 0.32 | 0.27 |  |  | 0.06 |  |  |
| Glycerol from biodiesel production | 95% (w/w) | MEAN |  |  |  |  |  |  |  |  | [38] |
| Oil waste |  | MEAN |  |  |  | 0.08 | 0.01 | 0.91 |  |  | [68] |
| Other poultry solid manure |  | MEAN | 0.24 | 0.10 | 0.05 |  |  |  | 0.08 |  | [143] |
| Black liquor |  | MEAN | 0.45 | 0.22 | 0.28 |  | 0.02 |  | 0.00 |  | [122] |

^a^ good satisfying sufficient deficient not reliable, for details see Table 1

^b^ Of all waste wood, 79% belong to waste category AI and AII (i.e., untreated or mechanically-treated waste wood and treated, but free of wood preservatives and halogens, respectively) and can be considered feasible as feedstock due to quality issues [55].

^c^ For oil mill residues, only residues from rapeseed oil (press cake and press meal) were considered. Rape cultivation is predominant in Germany, accounting for 94% of total vegetable oil production [109].

^d^ For stalks from roadside, a survey of the German Federal Highway Research Institute was analyzed [92], and for the composition of found green wastes, additional studies were considered [89-91]

**
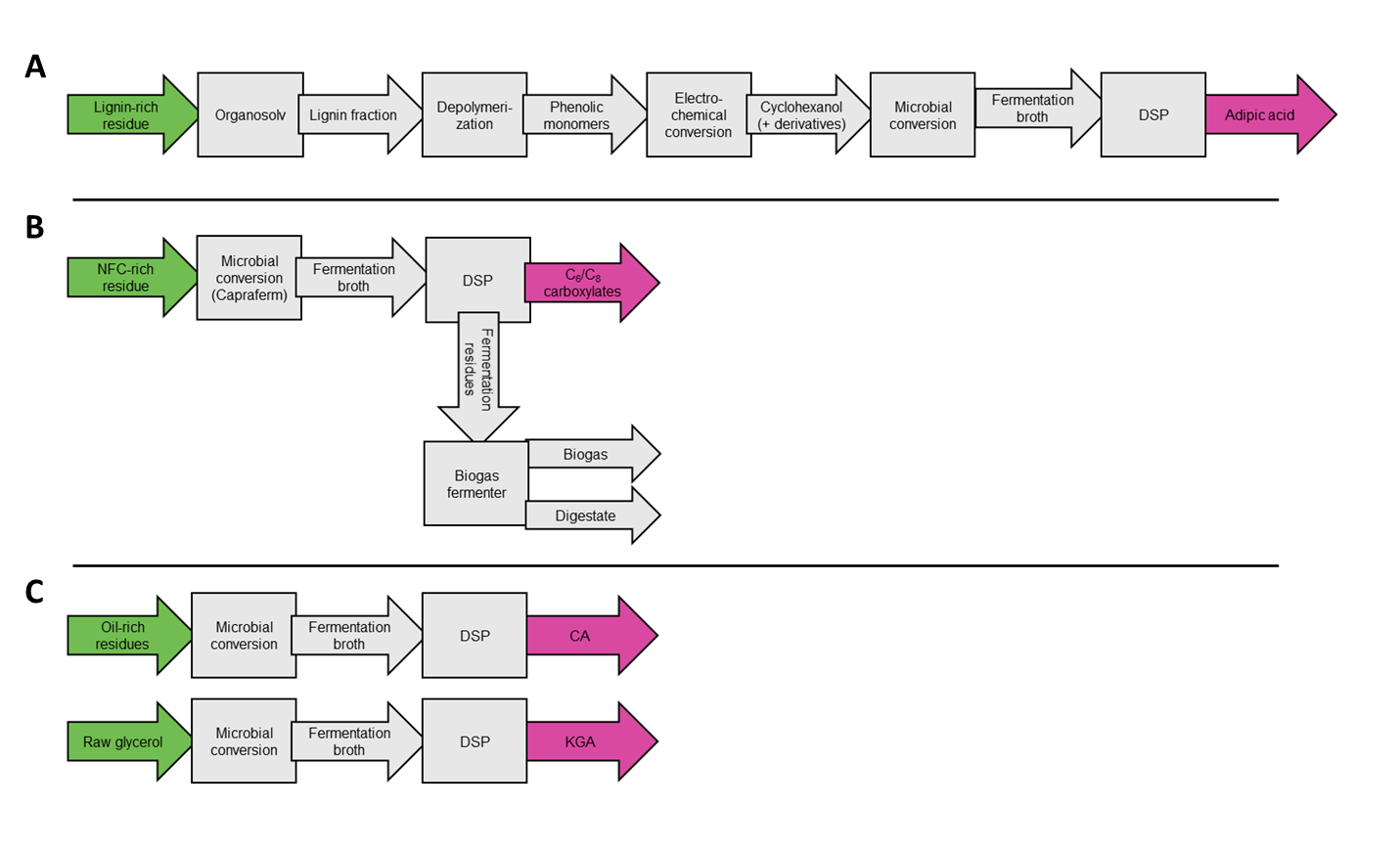
**

Figure S1: **Flowcharts illustrating the model processes based on biogenic residues. A**: Polymer bricks from lignin, **B**: Carboxylic acids from NFC, **C**: TCA cycle intermediates from waste cooking fat and oil. The depicted flowchart shows possible routes to utilize biogenic residues in selected biotechnological processes. DSP – downstream processing, CA – Citric acid, KGA – α-ketoglutaric acid.

**
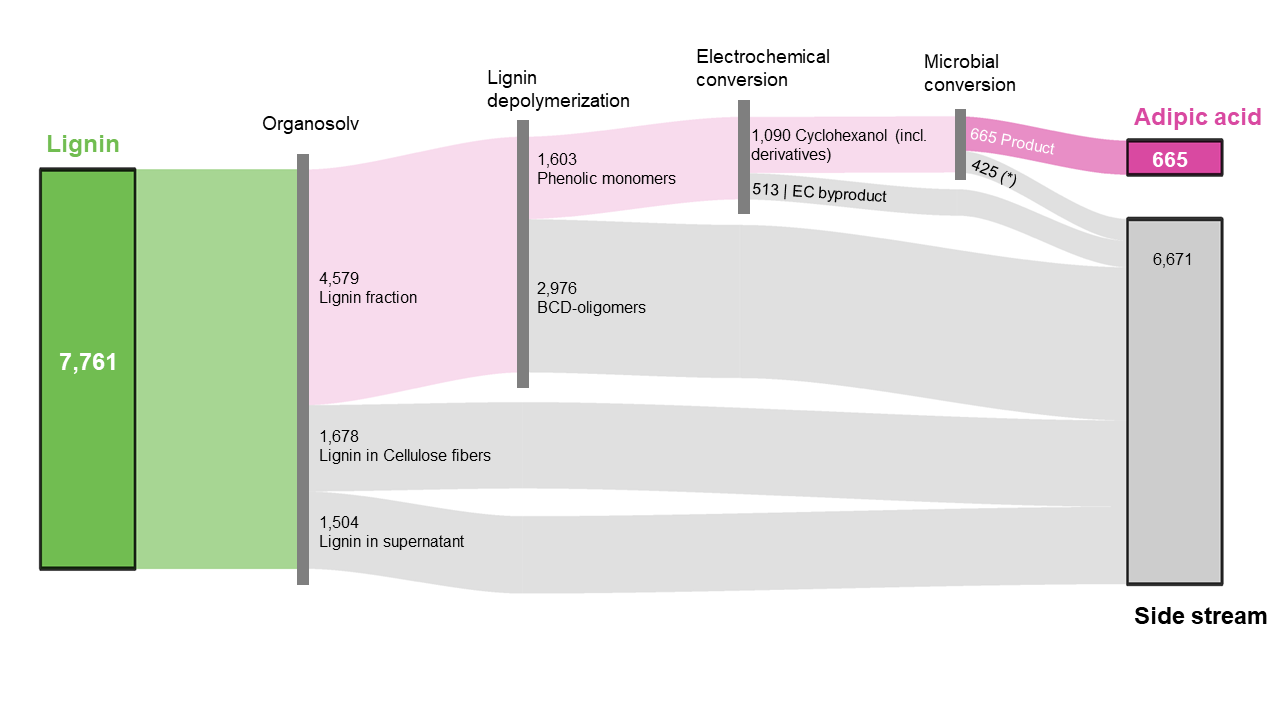
**

Figure S2: **Detailed material flow for the production of polymer brick adipic acid from lignin.** The lignin fraction needs to be separated in an organosolv process [22] and depolymerized [43] to be electrochemically and microbially converted to adipic acid [25]. (*) By-product of microbial process; BCD-based catalyzed depolymerization; EC – Electrochemical conversion. Numbers in 1,000 Mg a^-1^.

**References**

4. Thrän D, Brosowski A, Dotzauer MH, K., Hennig C, Herrmann A, Holzhammer U, Kalcher J, Kornatz P, Lenz V, Mast TN, S. *et al*. Genereller Rahmen & Definitionen. In: Methodenhandbuch: Stroffstromorientierte Bilanzierung der Klimagaseffekte. Edited by Thrän D, Pfeiffer D, vol. 4. Leipzig, Germany; 2021.

15. Batidzirai B, Smeets EMW, Faaij APC. Harmonising bioenergy resource potentials—Methodological lessons from review of state of the art bioenergy potential assessments. Renew Sustain Energy Rev 2012, 16(9):6598-6630.

18. Antal MJ, Allen SG, Dai X, Shimizu B, Tam MS, Grønli M. Attainment of the theoretical yield of carbon from biomass. Ind Eng Chem Res 2000, 39(11):4024-4031.

19. Naik S, Goud VV, Rout PK, Jacobson K, Dalai AK. Characterization of Canadian biomass for alternative renewable biofuel. Renew Energy 2010, 35(8):1624-1631.

21. Marktanalyse nachwachsende Rohstoffe [<https://www.fnr.de/marktanalyse/marktanalyse.pdf>]

22. Nitzsche R, Gröngröft A, Köchermann J, Meisel K, Etzold H, Verges M, Leschinsky M, Bachmann J, Saake B, Torkler S *et al*. Platform and fine chemicals from woody biomass: demonstration and assessment of a novel biorefinery. Biomass Convers Biorefin 2021, 11(6):2369-2385.

25. Morejón MC, Franz A, Karande R, Harnisch F. Integrated electrosynthesis and biosynthesis for the production of adipic acid from lignin-derived phenols. Green Chem 2023, 25(12):4662-4666.

38. Otto C, Yovkova V, Aurich A, Mauersberger S, Barth G. Variation of the by-product spectrum during α-ketoglutaric acid production from raw glycerol by overexpression of fumarase and pyruvate carboxylase genes in Yarrowia lipolytica. Appl Microbiol Biotechnol 2012, 95(4):905-917.

39. Barrett DG, Yousaf MN. Poly(triol α-ketoglutarate) as biodegradable, chemoselective, and mechanically tunable elastomers. Macromolecules 2008, 41(17):6347-6352.

43. Unkelbach G. Untersuchung zur Gewinnung von Lignin mittels autokatalytischem Ethanol/Wasser-Aufschluss und dessen hydrothermale Spaltung zu Phenolen. Universtität Stuttgart; 2021.

47. Frankó B, Galbe M, Wallberg O. Influence of bark on fuel ethanol production from steam-pretreated spruce. Biotechnol Biofuel 2015, 8(1):15.

48. Fradinho DM, Neto CP, Evtuguin D, Jorge FC, Irle MA, Gil MH, de Jesus JP. Chemical characterisation of bark and of alkaline bark extracts from maritime pine grown in Portugal. Industr Crop Prod 2002, 16(1):23-32.

49. Waliszewska B, Sieradzka A, Spek-Dźwigała A, Brózdowski J. Chemical composition of beech bark stripped and not stripped by animals. 2018, 104:420-425.

50. Jin W, Tingi K, Zondlo J, Wang J, Brar J: Pyrolysis kinetics of physical components of wood and wood-polymers using isoconversion method, vol. 3; 2012.

51. Stiller AH, Dadyburjor DB, Wann J, Tian D, Zondlo JW. Co-processing of agricultural and biomass waste with coal. FPT 1996, 49(1):167-175.

52. Rusanen A, Lappalainen K, Kärkkäinen J, Tuuttila T, Mikola M, Lassi U. Selective hemicellulose hydrolysis of Scots pine sawdust. Biomass Convers Biorefin 2019, 9(2):283-291.

53. Kangas H, Felissia, Filgueira D, Ehman N, Vallejos M, Imlauer, Lahtinen, Area M, Chinga Carrasco G. 3D printing high-consistency enzymatic nanocellulose obtained from a soda-ethanol-O_2_ pine sawdust pulp. Bioengineering 2019, 6:60.

54. Boadu KB, Nsiah-Asante R, Antwi RT, Obirikorang KA, Anokye R, Ansong M. Influence of the chemical content of sawdust on the levels of important macronutrients and ash composition in Pearl oyster mushroom (*Pleurotus ostreatus*). PLOS ONE 2023, 18(6):e0287532.

55. Thrän D, Kaltschmitt, M., Siegmund, T., Karras, T. Nebenprodukte, Rückstände und Abfälle. In: Energie aus Biomasse - Ressourcen und Bereitstellung. Edited by Kaltschmitt MS, K., vol. 4, 4 edn: Springer Verlag; 2024.

56. Mussatto SI, Dragone G, Roberto IC. Brewers' spent grain: generation, characteristics and potential applications. J Cereal Sci 2006, 43(1):1-14.

57. Le Floch A, Jourdes M, Teissedre P. Polysaccharides and lignin from oak wood used in cooperage: Composition, interest, assays: A review. Carbohydr Res 2015, 417:94-102.

58. Laskowska AM, M., Boruszewski PW, J. Chemical composition and selected physical properties of oak wood (*Quercus robur L.*) modified by cyclic thermo-mechanical treatment. Bioresources 2018, 13(4):9005-9019.

59. Cárdenas-Gutiérrez M, Pedraza-Bucio F, Lopez-Albarran P, Rutiaga-Quinones JG, Correa-Méndez F, Carrillo-Parra A, Herrera-Bucio R. Chemical components of the branches of six hardwood species. Wood Res 2018, 63(5):795-808.

60. Ruxanda B, Teacă C, Spiridon I. Chemical modification of beech wood: Effect on thermal stability. Bioresources 2008, 3.

61. Bari E, Taghiyari HR, Mohebby B, Clausen CA, Schmidt O, Tajick Ghanbary MA, Vaseghi MJ. Mechanical properties and chemical composition of beech wood exposed for 30 and 120 days to white-rot fungi. Holzforschung 2014, 69(5):587-593.

62. Čabalová I, Bélik M, Kučerová V, Jurczyková T. Chemical and morphological composition of Norway spruce wood (*Picea abies, L.*) in the dependence of its storage. Polymers 2021, 13(10).

63. Filipova I, Grinfelds U, Jansons A, Andze L, Irbe I, Verovkins A, Treimanis A. Comparison of the properties of wood and pulp fibers from lodgepole pine (*Pinus contorta*) and scots pine (*Pinus sylvestris*). Bioresources 2012, 7:1771-1783.

64. Liu X, Xie Y, Sheng H. Green waste characteristics and sustainable recycling options. Resour Environ Sustain 2023, 11:100098.

65. Eduktdatenblatt Grüngut [<https://www.dbfz.de/fileadmin/Pilot_SBG/Eduktdatenblaetter/Gruengut-Edukt-Datenblattreihe-Biogasgewinnung-2021-2022-Leipzig-DBFZ-Pilot-SBG-07092022.pdf>]

66. Cortez J, Demard JM, Bottner P, Jocteur Monrozier L. Decomposition of mediterranean leaf litters: A microcosm experiment investigating relationships between decomposition rates and litter quality. Soil Biol Biochem 1996, 28(4):443-452.

67. Fourty T, Baret F, Jacquemoud S, Schmuck G, Verdebout J. Leaf optical properties with explicit description of its biochemical composition: Direct and inverse problems. RSE 1996, 56(2):104-117.

68. Slopiecka K, Liberti F, Massoli S, Bartocci P, Fantozzi F. Chemical and physical characterization of food waste to improve its use in anaerobic digestion plants. Energy Nexus 2022, 5:100049.

69. Gaida B, Schüttmann I, Zorn H, Mahro B. Bestandsaufnahme zum biogenen Reststoffpotential der deutschen Lebensmittel- und Biotechnik-Industrie. In. Fachagentur Nachwachsende Rohstoffe; 2013.

70. Mayer F, Hillebrandt J. Potato pulp: microbiological characterization, physical modification, and application of this agricultural waste product. Appl Microbiol Biotechnol 1997, 48(4):435-440.

71. Klingspohn U, Bader J, Kruse B, Vijai Kishore P, Schügerl K, Kracke-Helm HA, Likidis Z. Utilization of potato pulp from potato starch processing. Process Biochem 1993, 28(2):91-98.

72. Tommaso G, Ribeiro R, de Oliveira CAF, Stamatelatou K, Antonopoulou G, Lyberatos G, Hodúr C, Csanádi J. Clean strategies for the management of residues in dairy industries. In: Novel Technologies in Food Science: Their Impact on Products, Consumer Trends and the Environment. Edited by McElhatton A, do Amaral Sobral PJ. New York, NY: Springer New York; 2012: 381-411.

73. Shinde G, Kumar R, Chauhan S, Subramanian V, Nadanasabapathi S. Whey Proteins: A potential ingredient for food industry- A review. Asian JDFR 2018.

74. Meisel K, Braune M, Gröngröft A, Majer S, Müller-Langer F, Naumann K, Oehmichen K. Technische und methodische Grundlagen der THG-Bilanzierung von Bioethanol. DBFZ Handreichung 2015.

75. Concha Olmos J, Zúñiga Hansen ME. Enzymatic depolymerization of sugar beet pulp: Production and characterization of pectin and pectic-oligosaccharides as a potential source for functional carbohydrates. Chem Eng 2012, 192:29-36.

76. Pińkowska H, Krzywonos M, Wolak P, Złocińska A. Pectin and neutral monosaccharides production during the simultaneous hydrothermal extraction of waste biomass from refining of sugar - optimization with the use of Doehlert design. Molecules 2019, 24:472.

77. Singh K, Honig H, Wermke M, Zimmer E. Fermentation pattern and changes in cell wall constituents of straw-forage silages, straws and partners during storage. AFST 1996, 61(1):137-153.

78. Sjölin M, Thuvander J, Wallberg O, Lipnizki F. Purification of sucrose in sugar beet molasses by utilizing ceramic nanofiltration and ultrafiltration membranes. Membranes 2019, 10:5.

79. Malakahmad A, Basri N, Zain S: Production of renewable energy by transformation of kitchen waste to biogas, case study of Malaysia; 2011.

80. Tang Y-Q, Koike Y, Liu K, An M-Z, Morimura S, Wu X-L, Kida K. Ethanol production from kitchen waste using the flocculating yeast *Saccharomyces cerevisiae* strain KF-7. Biomass Bioenergy 2008, 32(11):1037-1045.

81. Yu M, Zhao M, Huang Z, Xi K, Shi W, Ruan W. A model based on feature objects aided strategy to evaluate the methane generation from food waste by anaerobic digestion. Waste Manag 2018, 72:218-226.

82. Vavouraki AI, Angelis EM, Kornaros M. Optimization of thermo-chemical hydrolysis of kitchen wastes. Waste Manag 2013, 33(3):740-745.

83. Cortez LAB, Baldassin R, de Almeida E. Chapter 7 - Energy from sugarcane. In: Sugarcane Biorefinery, Technology and Perspectives. Edited by Santos F, Rabelo SC, De Matos M, Eichler P: Academic Press; 2020: 117-139.

84. Li R, Chen S, Li X, Saifullah Lar J, He Y, Zhu B. Anaerobic co-digestion of kitchen waste with cattle manure for biogas production. Energy Fuels 2009, 23(4):2225-2228.

85. Shen J, Zhao C, Liu Y, Zhang R, Liu G, Chen C. Biogas production from anaerobic co-digestion of durian shell with chicken, dairy, and pig manures. Energy Convers Manag 2019, 198:110535.

86. Zhao Y, Sun F, Yu J, Cai Y, Luo X, Cui Z, Hu Y, Wang X. Co-digestion of oat straw and cow manure during anaerobic digestion: Stimulative and inhibitory effects on fermentation. Bioresour Technol 2018, 269:143-152.

87. Hilgert JE, Herrmann C, Petersen SO, Dragoni F, Amon T, Belik V, Ammon C, Amon B. Assessment of the biochemical methane potential of in-house and outdoor stored pig and dairy cow manure by evaluating chemical composition and storage conditions. Waste Manag 2023, 168:14-24.

88. Li K, Liu R, Sun C. Comparison of anaerobic digestion characteristics and kinetics of four livestock manures with different substrate concentrations. Bioresour Technol 2015, 198:133-140.

89. Bary AI, Cogger CG, Sullivan DM, Myhre EA. Characterization of fresh yard trimmings for agricultural use. Bioresour Technol 2005, 96(13):1499-1504.

90. Waliszewska B, Grzelak M, Gaweł E, Spek-Dźwigała A, Sieradzka A, Czekala W. Chemical characteristics of selected grass species from Polish meadows and their potential utilization for energy generation purposes. Energies 2021, 14:1669.

91. Krenz LMM, Pleissner D. Valorization of landscape management grass. Biomass Convers Biorefin 2024, 14(3):2889-2905.

92. Rommeiß N, Thrän, D., Schlägl, T., Daniel, J., Scholwin, F. Energetische Verwertung von Grünabfallen aus dem Straßenbetriebsdienst. In: Berichte der Bundesanstalt für Straßenwesen. vol. V 150. Bergisch Gladbach, Germany; 2006.

93. Viretto A, Gontard N, Angellier-Coussy H. Urban parks and gardens green waste: A valuable resource for the production of fillers for biocomposites applications. Waste Manag 2021, 120:538-548.

109. BLE. Bericht zur Markt- und Versorgungslage - Ölsaaten, Öle und Fette. In. Edited by Ernährung BfLu. Bundesanstalt für Landwirtschaft und Ernährung; 2023.

113. Phyllis 2: Database for biomass and waste [<https://phyllis.nl/>]

114. Schmidt T, Schneider F, Claupein E. Food waste in private households in Germany. In: Thünen Working Paper. vol. 92a. Braunschweig, Germany; 2019.

115. Eduktdatenblatt Rindergülle [<https://www.dbfz.de/fileadmin/Pilot_SBG/Eduktdatenblaetter/Rinderguelle-Edukt-Datenblattreihe-Biogasgewinnung-2021-2022-Leipzig-DBFZ-Pilot-SBG-07092022.pdf>]

116. Eduktdatenblatt Biogut [<https://www.dbfz.de/fileadmin/Pilot_SBG/Eduktdatenblaetter/Biogut-Edukt-Datenblattreihe-Biogasgewinnung-2021-2022-DBFZ-Pilot-SBG-Leipzig-05092022_01.pdf>]

117. Adamović M, Grubić G, Milenković I, Jovanović R, Protić R, Sretenović L, Stoićević L. The biodegradation of wheat straw by *Pleurotus ostreatus* mushrooms and its use in cattle feeding. AFST 1998, 71(3):357-362.

118. Bledzki AK, Mamun AA, Volk J. Physical, chemical and surface properties of wheat husk, rye husk and soft wood and their polypropylene composites. Composites Part A: Applied Science and Manufacturing 2010, 41(4):480-488.

119. Lynd LR, Wyman CE, Gerngross TU. Biocommodity Engineering. Biotechnol Prog 1999, 15(5):777-793.

120. Bozkurt M, Alçiçek A, Cabuk M. The effect of dietary inclusion of meat and bone meal on the performance of laying hens at old age. S Afr J Anim Sci 2004, 34.

121. Montane D, Farriol X, Salvadó J, Jollez P, Chornet E. Application of steam explosion to the fractionation and rapid vapor-phase alkaline pulping of wheat straw. Biomass and Bioenergy 1998, 14(3):261-276.

122. Cardoso M, de Oliveira ÉD, Passos ML. Chemical composition and physical properties of black liquors and their effects on liquor recovery operation in Brazilian pulp mills. Fuel 2009, 88(4):756-763.

123. Cherney JH, Johnson KD, Volenec JJ, Anliker KS. Chemical composition of herbaceous grass and legume species grown for maximum biomass production. Biomass 1988, 17(4):215-238.

124. Nigam JN. Ethanol production from wheat straw hemicellulose hydrolysate by *Pichia stipitis*. J Biotechnol 2001, 87(1):17-27.

125. Choi H, Won CS, Kim BG. Protein and energy concentrations of meat meal and meat and bone meal fed to pigs based on *in vitro* assays. Anim Nutr 2021, 7(1):252-257.

126. Ong HK, Pullammanappallil PC, Greenfield PF. Physical, chemical and biomethanation characteristics of stratified cattle-manure slurry. Asian-Australas J Anim Sci 2000, 13(11):1593-1597.

127. Sander B. Properties of Danish biofuels and the requirements for power production. Biomass and Bioenergy 1997, 12(3):177-183.

128. Shen F, Zhong B, Wang Y, Xia X, Zhai Z, Zhang Q. Cellulolytic microflora pretreatment increases the efficiency of anaerobic co-digestion of rice straw and pig manure. BioEnergy Research 2019, 12(3):703-713.

129. Verhe R, Varghese S, Thevelein JM, Nikroo JH, Lambrecht M, Redant E, De Clerq G. Production of bio-ethanol from the organic fraction of municipal solid wase and refuse-derived fuel. biomass 2022, 2:224-236.

130. Lee J. Biological conversion of lignocellulosic biomass to ethanol. J Biotech 1997, 56(1):1-24.

131. Leming R, Lember A. Chemical composition of expeller-extracted and cold-pressed canola meal. Agraarteadus 2005, 16.

132. Zhou X, Beltranena E, Zijlstra RT. Effects of feeding canola press-cake on diet nutrient digestibility and growth performance of weaned pigs. AFST 2016, 211:208-215.

133. Li R, Tan W, Zhao X, Dang Q, Song Q, Xi B, Zhang X. Evaluation on the methane production potential of wood waste pretreated with NaOH and co-digested with pig manure. Catalysts 2019, 9(6):539.

134. Smith JL, Garcia-Perez M, Das KC. Producing fuel and specialty chemicals from the slow pyrolysis of poultry DAF skimmings. JAAP 2009, 86:115-121.

135. Gandam PK, Chinta ML, Gandham AP, Pabbathi NPP, Konakanchi S, Bhavanam A, Atchuta SR, Baadhe RR, Bhatia RK. A new insight into the composition and physical characteristics of corncob: Substantiating its potential for tailored biorefinery objectives. Fermentation 2022, 8(12):704.

136. Kiritsakis AK: Olive oil: from the tree to the table: Food & Nutrition Press; 1998.

137. Nazifa TH, Saady NMC, Bazan C, Zendehboudi S, Aftab A, Albayati TM. Anaerobic digestion of blood from slaughtered livestock: A review. Energies 2021, 14(18):5666.

138. Zhou W, Gong Z, Zhang L, Liu Y, Yan J, Zhao M. Feasibility of lipid production from waste paper by the oleaginous yeast *Cryptococcus curvatus*. BioResources 2017, 12:5249-5263.

139. Bodennutzung und pflanzliche Erzeugung - Getreide [<https://www.bmel-statistik.de/landwirtschaft/bodennutzung-und-pflanzliche-erzeugung/getreide>]

140. Garcia Gonzalez MN, Björnsson L. Life cycle assessment of the production of beet sugar and its by-products. J Clean Prod 2022, 346:131211.

141. Li Y, Zhang R, Chen C, Liu G, He Y, Liu X. Biogas production from co-digestion of corn stover and chicken manure under anaerobic wet, hemi-solid, and solid state conditions. Bioresour Technol 2013, 149:406-412.

142. Bindu JP, Ramakrishna Parama VR, Srinivasamurthy CA. Changes in nutrient and biochemical constituents on decomposition of leaf litter of selected tree species. Mysroe J Agric Sci 2014, 48(3):364-373.

143. Rahman MA, Møller HB, Saha CK, Alam MM, Wahid R, Feng L. Anaerobic co-digestion of poultry droppings and briquetted wheat straw at mesophilic and thermophilic conditions: Influence of alkali pretreatment. Renewable Energy 2018, 128:241-249.
